# Clinician Trust and Human Factors in AI-Enabled Clinical Decision Support in Acute Care: Mixed Methods Study

**DOI:** 10.2196/95472

**Published:** 2026-10-02

**Authors:** Meghana Darla, Danielle Miltz, Khushboo Chandnani, Saptarshi Purkayastha, John W Diehl, Sivasubramanium V Bhavani

**Affiliations:** 1 Department of Otolaryngology, School of Medicine, Emory University, Emory University Hospital Midtown, 550 Peachtree St NE, Suite 1135, Atlanta, GA, 30324, United States, 1 9087580993; 2 Department of Biomedical Engineering and Informatics, Luddy School of Informatics, Computing, and Engineering, Indiana University, Indianapolis, IN, United States; 3 Surgical Transplant Intensive Care Unit, Emory Critical Care Center, Emory Healthcare, Atlanta, GA, United States; 4 Department of Emergency Medicine, School of Medicine, Emory University, Atlanta, GA, United States; 5 Department of Medicine, School of Medicine, Emory University, Atlanta, GA, United States

**Keywords:** clinical decision support systems, artificial intelligence, critical care, human factors, clinician surveys, trust in automation, technology acceptance model, SEIPS model, Systems Engineering Initiative for Patient Safety

## Abstract

**Background:**

AI has the potential to enhance clinical decision-making in high-acuity settings such as intensive care units (ICUs) and emergency departments (EDs). However, despite promising performance, many AI-driven clinical decision support systems (AI-CDSSs) face poor adoption due to issues of trust, workflow disruption, and alert fatigue. Understanding the human factors that shape clinician acceptance is critical to guide safe and effective implementation of AI-CDSS in acute care. Theoretical frameworks, including the Systems Engineering Initiative for Patient Safety (SEIPS) 2.0 model and the technology acceptance model (TAM), suggest that successful adoption requires addressing sociotechnical interactions among clinician trust, system design, organizational readiness, and task complexity, yet few empirical studies have applied these frameworks to AI-CDSSs in acute care settings.

**Objective:**

This study aimed to evaluate emergency medicine and critical care clinicians’ perceptions of AI-CDSSs and to identify key factors influencing adoption, including trust, design preferences, and workflow integration.

**Methods:**

A SEIPS 2.0–informed mixed methods study evaluated ICU and ED clinicians from Emory Healthcare on perceptions of AI in clinical practice. An expert-reviewed survey (N=57) assessed clinician perceptions, trust, and implementation preferences. Semistructured interviews (n=11) included A/B testing of AI-CDSSs and clinical sepsis scenarios to explore decision-making in context. Transcripts were thematically analyzed using the Braun and Clarke framework in ATLAS.ti (version 26, ATLAS.ti Scientific Software Development). Quantitative data were analyzed descriptively. This study assessed clinician perceptions using mock alerts and hypothetical scenarios rather than real-world AI-CDSS deployment.

**Results:**

Trust in AI varied significantly by patient acuity (Cochran Q=30.40, *P*<.001): stable patients (43/57, 75%, 95% CI 63%‐85%), deteriorating patients (27/57, 47%, 95% CI 35%‐60%), and critically ill patients in the ICU and undifferentiated patients in the ED (25/57, 44%, 95% CI 32%‐57% for each scenario). Internal consistency was acceptable-to-good across three scales (Cronbach α: AI Perception=.891, Trust=.743, Implementation=.740; McDonald *ω*: AI Perception=0.895, Trust=0.782, Implementation=0.746). Barriers included overreliance, insufficient training, and data quality concerns. For the exploratory AI-CDSS design, clinicians preferred opt-in alerts (10/11, 91%), evidence-linked recommendations (7/11, 64%), and avoiding overt mention of increased AI acceptance (8/11, 73%). Thematic analysis yielded 36 themes across six domains: trust and transparency, alert usability, workflow fit, data concerns, training needs, and perceived clinical impact. Clinicians favored AI-CDSSs that preserved autonomy, minimized disruption, and provided transparent rationales.

**Conclusions:**

Adoption of AI-CDSSs in critical care is not solely a technical issue but a human-factors challenge centered on trust, transparency, and workflow compatibility. These findings support future testing of a phased implementation approach—beginning with lower-acuity applications where clinician trust is highest, then gradually extending to higher-acuity scenarios with enhanced transparency and override mechanisms. This graduated strategy addresses the critical interdependencies among people (trust), tools (design), organizations (training), and tasks (clinical complexity) identified in this study.

## Introduction

The integration of AI tools into clinical practice offers an opportunity to enhance health care delivery, particularly in high-acuity settings like the intensive care unit (ICU) and emergency department (ED), where rapid, data-driven decisions are crucial [1]. Despite the technological promise, the successful implementation of AI tools into frontline clinical practice is filled with challenges [2]. The history of health information technology is replete with examples of powerful tools that failed to gain traction due to a mismatch with clinical workflow. This mismatch is often attributed to a lack of attention to “human factors,” which refer to the complex interplay between technology, users, and the clinical environment [3]. The adoption of AI is similarly challenged by significant barriers, including a lack of trust, concerns about workflow disruption, and the potential for alert fatigue [3-5]. Overcoming these barriers requires a deep understanding of clinician perceptions, needs, and priorities.

A clinical decision support system (CDSS) is a practical application that can integrate AI into clinical decision-making [4]. A targeted intervention can be suggested to the clinician through a CDSS from a validated machine learning algorithm. For instance, in the Precision Resuscitation With Crystalloids in Sepsis (PRECISE) trial, a validated machine learning algorithm identifies a subgroup of infected patients with a potential mortality benefit from balanced crystalloid instead of normal saline, and a CDSS prompts the clinician to change the order [6]. Since the integration of CDSSs into electronic health records (EHRs), there have been multiple studies on the “five rights” of CDSSs, which help guide the creation of effective alerts [7]; however, less is understood on how to effectively incorporate a CDSS that utilizes an AI recommendation. Because successful AI-based CDSS (AI-CDSS) implementation depends on more than the technical performance of the algorithm, a human factors framework is needed to examine how clinician trust, workflow, alert design, and organizational context shape adoption [8,9].

Using multiple complementary frameworks to evaluate implementation of an AI tool can provide a more holistic understanding. We primarily draw on the Systems Engineering Initiative for Patient Safety (SEIPS) 2.0 model [10], which conceptualizes health care systems as comprising five interacting components: people, tasks, tools and technologies, organization, and environment. SEIPS 2.0 emphasizes that patient safety and quality outcomes emerge from complex, bidirectional interactions among these components, making it particularly suitable for studying the adoption of AI-CDSSs, which simultaneously affects clinician cognition (people), decision-making workflows (tasks), interface design requirements (tools), implementation strategy (organization), and care setting constraints (environment). We also incorporate constructs from the technology acceptance model (TAM) [11], specifically Perceived Usefulness and Perceived Ease of Use, and Jian et al’s [12] Trust in Automated Systems framework, which characterizes trust across belief, intention, and behavior dimensions. In addition, we draw on the three-layer trust model of Hoff and Bashir [13], which distinguishes dispositional, situational, and learned trust and offers an integrative lens for trust in automation. This multiframework approach responds to calls for theoretically grounded, human-centered evaluations of health care AI [8,9].

In this study, we address the gap in knowledge on applying AI-CDSSs to high acuity settings through a mixed methods evaluation to investigate ED and ICU clinicians’ perceptions of AI use in clinical practice and to identify and analyze the facilitators and barriers to AI adoption.

## Methods

### Study Design

A mixed methods human factors evaluation was conducted at Emory Healthcare in Atlanta, Georgia. The study employed a cross-sectional, exploratory design and combined a quantitative survey with qualitative, semistructured interviews. We used a convergent (concurrent triangulation) design in which the quantitative and qualitative strands were collected in parallel and given equal priority (QUAN+QUAL). Integration occurred at the methods level, through shared constructs across the survey and interview guide, and at the interpretation level, through a joint display. The mixed methods reporting follows the Good Reporting of a Mixed Methods Study (GRAMMS) criteria [14] (Checklist 1). SEIPS 2.0 also guided the study operationally across data collection, analysis, and interpretation. Survey domains and the semistructured interview guide were developed to reflect SEIPS 2.0 work-system components, including people, tasks, tools and technologies, organization, and environment. These domains were later used as sensitizing concepts during codebook refinement, theme development, and interpretation. The objective was to explore clinician perceptions of AI-CDSSs.

### Recruitment and Participants

Eligible participants included clinicians in the ICU and ED, who were actively involved in direct patient care. Recruitment was conducted from February to April 2025 through multiple channels: invitation emails distributed via ICU and ED provider listservs at all Emory Healthcare hospitals, institutional review board (IRB)–approved flyers posted in clinical units, presentations during departmental meetings, and word-of-mouth referrals among clinical staff. Flyers included a QR code directing interested individuals to the online REDCap survey platform. Participants could opt to complete the survey, participate in an interview, or both. Participation was voluntary and could be discontinued at any point.

### Data Collection

#### Survey Procedures

The survey instrument was developed through an iterative consensus process with subject matter experts in critical care and health informatics. The instrument assessed four primary domains: (1) clinician demographics and prior experience with AI, (2) general perceptions of AI’s role and impact, (3) trust in AI recommendations across different clinical scenarios, and (4) the importance of various AI usability features. The survey was administered online via REDCap (Vanderbilt University) and remained open from February 9 to April 1, 2025. Items used Likert-scale (5-point), multiple-choice, and open-ended response formats.

The survey was voluntary and distributed through ICU and ED provider listservs, IRB-approved flyers with QR codes, departmental presentations, and word-of-mouth referral. The survey was administered electronically through REDCap, and participants reviewed study information and provided informed consent before participation. No survey weighting was applied. Since recruitment occurred through both listserv-based and open-ended channels, the exact number of clinicians who received or viewed the invitation could not be determined, and a precise response rate could not be calculated. A Checklist for Reporting Results of Internet E-Surveys (CHERRIES) checklist was completed [15] (Checklist 2).

The survey items were organized into four construct groups for analysis: (1) AI perception (6 items mapping to TAM perceived usefulness: accuracy, efficiency, variability, the two complementarity items, and reliability; the negatively worded overreliance item was analyzed as a standalone item), (2) trust scenarios (4 binary items across patient acuity levels, informed by Jian et al’s [12] trust-in-automation framework), (3) trust beliefs (3 Likert items: reliability, understanding-trust link, complementarity), and (4) implementation factors (6 importance-rated items mapping to unified theory of acceptance and use of technology [UTAUT]; facilitating conditions and TAM perceived ease of use [11,16]).

#### Interview Procedures

Clinicians who completed the survey were subsequently invited to participate in semistructured interviews conducted via Zoom (Zoom Communications, Inc) from March 20 to April 7, 2025. Interviews lasted approximately 30 to 45 minutes and followed a structured guide developed by the research team based on existing literature regarding AI-CDSS adoption. All interviews were conducted by the lead analyst (MD, who has clinical training in dentistry and informatics). The interview component is reported in accordance with the Consolidated Criteria for Reporting Qualitative Research (COREQ) [17] (Checklist 3). Interview questions explored the same domains as the survey while allowing for deeper examination of participants’ reasoning and preferences. The interviews incorporated A/B testing of different AI-CDSS interface designs. Alert prototypes were derived from the AI-CDSS utilized in the PRECISE trial, which encourages clinicians to consider changing their order from normal saline to balanced crystalloid in a population of infected patients that have been shown to have a mortality benefit with balanced crystalloid using an AI algorithm [6]. Participants evaluated three distinct pairs of mock-up alerts (version A vs version B; Multimedia Appendices 1–4), each designed to assess specific aspects of usability and information framing. For each pair, participants selected their preferred design and articulated detailed reasoning for their choice. The presentation order of A/B test pairs was consistent across participants; randomization or counterbalancing was not employed, which may introduce order effects. Additionally, each A/B pair tested multiple design variables simultaneously (eg, opt-in vs opt-out combined with different action language), limiting the ability to isolate individual design effects.

Participants were also presented with a clinical case scenario designed to elicit nuanced perspectives on the practical application of an AI-CDSS. Nurses were excluded from this section since the decisions made may be outside their scope of practice. The scenario depicted a 54-year-old male with multiple comorbidities presenting with septic shock. This case was used to anchor the discussion and probe opinions across five decision points in a critical care resuscitation (Multimedia Appendix 5).

All interviews were audio-recorded using Zoom’s built-in recording function, transcribed verbatim using automated transcription with subsequent manual review for accuracy, and deidentified prior to analysis. Complete survey and interview instruments are provided in Multimedia Appendices 6 and 7 respectively.

### Data Analysis

Survey responses were analyzed using descriptive statistics (frequencies, proportions, means, and SDs) computed in Python (SciPy, NumPy). Proportions are reported with 95% Wilson score CI, preferred over Wald intervals for small samples and extreme proportions. Likert-type items (5-point scales) are reported with means, SDs, and 95% CIs. Internal consistency of multi-item scales was assessed using Cronbach α. The McDonald *ω* (total, from a one-factor model) is analyzed alongside α because ω provides a less restrictive estimate of internal consistency and is recommended as a practical complement to α [18]. Trust across four patient acuity levels was compared using Cochran Q test for related binary samples, with pairwise McNemar exact tests and Bonferroni correction (6 comparisons, adjusted α=.0083). Holm step-down and Benjamini-Hochberg false discovery rate adjustments are presented alongside Bonferroni, and a Friedman test on the full 5-point trust ratings served as a nonparametric sensitivity analysis. Medians and IQRs are reported with means and SDs for Likert-type items in a supplementary table (Multimedia Appendix 8). Associations between categorical variables (department×perception, role×AI usage, AI experience×trust) were assessed using chi-square tests or Fisher exact tests when expected cell counts fell below 5. Since more than 20% of expected cell counts were below 5 in these cross-tabulations, they were treated as exploratory sensitivity analyses, and Monte Carlo permutation *P* values (fixed margins, 100,000 simulated tables) were computed alongside the asymptotic tests. Given the exploratory sample size (N=57), we report exact counts alongside percentages and note cell-count limitations for cross-tabulations. For the multiselect item on desired AI applications, respondents could choose any number of options, and percentages are reported as shares of all selections. Exploratory subgroup analyses compared ICU and ED respondents on each main outcome using 95% Wilson CIs, Fisher exact tests for binary outcomes, and Mann-Whitney *U* tests for ordinal items.

Interview transcripts and open-ended survey responses were analyzed using thematic analysis following the six-phase framework described by Braun and Clarke [19]. Two reviewers (MD and KC) independently coded the data using ATLAS.ti (version 26; ATLAS.ti Scientific Software Development) and Microsoft Excel. Coding was conducted both deductively, based on the structure of the interview guide, and inductively to capture emergent patterns. The reviewers first reviewed transcripts to identify recurring ideas, phrases, and concepts, which were then labeled as codes and organized into a shared codebook, through consensus discussions with a third reviewer (SP), refining the codebook across multiple coding cycles. During codebook refinement and theme development, codes and excerpts were reviewed in relation to SEIPS 2.0 work-system domains while allowing inductive themes to emerge from the data. Related codes were then collated into preliminary themes and subthemes based on recurring patterns in the interview transcripts. These preliminary themes were reviewed against the underlying excerpts, refined through consensus discussions among the analytic team, and revised to reduce overlap and improve conceptual clarity. Themes addressing similar concepts were then merged into six overarching thematic clusters based on recurrence across responses, relevance to the study aims, clinical interpretability, and alignment with SEIPS 2.0 work-system domains. The final cluster structure was validated by comparing each cluster against representative excerpts, resolving discrepancies through coder discussion, and integrating the qualitative findings with survey results. Integration types in the joint display (convergent, complementary, expansion, or emergent) were assigned through consensus discussion between the two coders. Open-ended survey responses were coded using the same codebook to ensure analytical coherence across data sources. An audit trail was maintained throughout the analytic process, and regular debriefing sessions among coders ensured analytic rigor. Intercoder agreement was assessed at early coding stages using percentage agreement, which exceeded 85% prior to consensus discussion. A third reviewer (SP) not directly involved in the clinical trial adjudicated discrepancies.

### Ethical Considerations

The study received approval from the Emory University and Indiana University IRBs (IRB #STUDY00008904 and Protocol #26246 respectively), and all participants provided informed consent prior to participation and data collection. All study procedures adhered to ethical guidelines for human subjects’ research. All data were deidentified, and transcripts and survey data were stored on secure, encrypted servers. Participants’ identities were protected throughout data analysis and reporting.

We acknowledge that the positionality of the research team, several members of which are embedded in the PRECISE trial [6], may introduce bias toward favorable framing of AI-CDSSs. The lead analyst (MD) conducted interviews with prior knowledge of AI-CDSS design principles; however, the dual-coder approach, inclusion of clinician coinvestigators (DM, JWD, SVB) with direct patient care responsibilities, analytic debriefing, and team-based consensus review during analysis helped mitigate interpretive bias. We have attempted to present both supportive and critical clinician perspectives throughout.

## Results

### Survey Findings

#### Respondent Characteristics

Fifty-seven clinicians completed the survey (98.2% completion rate, with 1 partially incomplete). The sample comprised ICU providers (39/57, 68%, 95% CI 56%‐79%), ED providers (13/57, 23%, 95% CI 14%‐35%), and other settings or the general ward (5/57, 9%, 95% CI 4%‐19%). Respondents represented diverse clinical roles: ICU advanced practice providers (APP; 15/57, 26%), nurses (12/57, 21%), residents and fellows (9/57, 16%), intensivists (8/57, 14%), emergency physicians (7/57, 12%), and ED APPs, pharmacists, and other roles (6/57, 11%). Experience ranged from <1 year (2/57, 4%) to >15 years (9/57, 16%), with the largest group at 1‐5 years (18/57, 32%).

Of 57 respondents, 22 (39%, 95% CI 27%‐51%) reported prior AI use in clinical practice, while 35 (61%) had not. Among AI users (n=22), usage frequency was daily (n=5, 23%, 95% CI 10%‐43%), weekly (n=11, 50%, 95% CI 31%‐69%), monthly (n=3, 14%), and rarely (n=3, 14%). Confidence in AI recommendations among users was generally positive: confident (n=8, 36%, 95% CI 20%‐57%), moderately confident (n=9, 41%, 95% CI 23%‐61%), slightly confident (n=3, 14%), and not at all confident (n=2, 9%). Combined positive confidence (confident+moderately confident) was 77% (95% CI 57%‐90%).

#### Perception of AI Role and Impact

Fifty-four percent (31/57, 95% CI 42%‐67%) of survey respondents perceived AI as “helpful but supplementary,” 23% (13/57, 95% CI 14%‐35%) considered it “transformative and essential,” 19% (n=11) were “neutral/uncertain,” and only 4% (2/57, 95% CI: 1%‐12%) viewed AI as “potentially disruptive.” Trust in AI varied significantly with patient acuity (Cochran Q_3_=30.40; *P*<.001): stable ward patients elicited the highest combined trust (somewhat trust+strongly trust) at 75% (95% CI 63%‐84%), followed by deteriorating ward patients at 47% (95% CI 35%‐60%), undifferentiated ED patients at 44% (95% CI 32%‐57%), and critically ill ICU patients at 44% (95% CI 32%‐57%). Notably, strong distrust increased markedly with acuity: from 2% for stable patients to 7% for deteriorating patients to 12% for critically ill ICU patients. Overall, 68 % (95% CI 56%‐79%) of respondents expressed ethical concerns about AI in clinical decision-making. Regarding overreliance, 79% (95% CI 67%‐88%) agreed or strongly agreed that overreliance on AI poses risks to patient outcomes.

Likert-scale responses further characterized clinician attitudes: most clinicians agreed that AI enhances care efficiency (36/57, 63%) and complements clinical expertise (39/57, 68% to 43/57, 75%), while over three-quarters reported that understanding AI outputs increases trust (45/57, 79%). At the same time, concerns about overreliance on AI were common (45/57, 79%). Confidence in AI’s reliability was more moderate, with 47% (27/57) agreeing that AI provides reliable insights. Internal consistency was considered good for the 6-item AI Perception scale (Cronbach α=0.891; McDonald *ω*=.895) and acceptable for the 3-item Trust subscale (α=0.743; *ω*=.782) and the 6-item Implementation scale (α=0.740; *ω*=.746), all exceeding the 0.70 acceptability threshold. Detailed Likert-scale statistics are presented in Table 1.

**Table 1.** Clinician attitudes toward AI-based clinical decision support system (Likert-scale responses).

Statement	Mean (SD)	95% CI (mean)	Median (IQR)	Agree and strongly agree combined % (95% CI)
AI improves accuracy of decisions	3.54 (0.78)	3.34‐3.75	4 (3-4)	56 (43‐68)
AI enhances care efficiency	3.79 (0.80)	3.58‐4.00	4 (3-4)	63 (50‐75)
AI reduces decision variability	3.51 (0.78)	3.31‐3.71	4 (3-4)	54 (42‐67)
AI complements clinical expertise	3.74 (0.77)	3.54‐3.94	4 (3-4)	68 (56‐79)
AI insights complement expertise	3.86 (0.74)	3.67‐4.05	4 (4-4)	75 (63‐85)
AI provides reliable insights	3.35 (0.88)	3.12‐3.58	3 (3-4)	47 (35‐60)
Overreliance on AI poses risks	4.05 (0.91)	3.82‐4.29	4 (4-5)	79 (67‐88)
Understanding AI outputs increases trust	4.14 (0.97)	3.89‐4.39	4 (4-5)	79 (67‐88)

Inferential analysis revealed that trust varied significantly across patient acuity levels (Cochran Q_3_=30.40; *P*<.001). Pairwise McNemar tests with Bonferroni correction (6 comparisons, adjusted α=.0083) confirmed that trust in stable patients was significantly higher than in deteriorating (*P*<.001), ICU (*P*<.001), and ED (all *P*<.001) scenarios, while the three nonstable scenarios did not differ significantly from each other. Conclusions were unchanged when Holm step-down and Benjamini-Hochberg false discovery rate adjustments were used instead of Bonferroni. A Friedman test on the full 5-point trust ratings confirmed the acuity effect (*χ*²_3_=33.5; *P*<.001; Kendall W=0.20). In exploratory sensitivity analyses, the cross-tabulations had more than 20% of expected cell counts below 5 and are not confirmatory. The association between department and AI perception was significant under the asymptotic test (*χ*²_9_=17.4*; P*=.04) but was not robust under Monte Carlo permutation testing (*P*=.08). The association between role and AI usage was significant (*χ*²_7_=16.5*; P*=.02; permutation *P*=.01). Both findings require confirmation in larger samples. Fisher exact test for AI experience and trust in stable patients was not significant (OR 0.79; 95% CI 0.23-2.69; *P*=.76).

#### Barriers to AI Adoption

Among the 22 respondents with AI experience, insufficient training (n=12, 55%, 95% CI 35%‐73%) and lack of trust in AI recommendations (n=12, 55%, 95% CI 35%‐73%) were the most frequently cited barriers, followed by workflow disruption (n=7, 32%, 95% CI 16%‐53%) and other factors (n=4, 18%, 95% CI 7%‐39%). Only 2 respondents (9%, 95% CI 3%‐28%) reported no barriers. The values here are reported among AI users only, as barriers are most meaningful for those with direct experience. Given the small denominator (n=22), these proportions have wide CIs and should be interpreted cautiously.

#### Desired Features and Implementation Priorities

Respondents universally emphasized the importance of explainability: 75% (43/57) rated transparency as “very important” and 25% (14/57) as “important,” achieving 100% combined endorsement (95% CI 94%‐100%). Furthermore, 79% (45/57, 95% CI 67%‐88%) agreed or strongly agreed that understanding how AI generates its outputs increases their trust. Implementation factor importance ratings (on a 5-point scale from “Not important” to “Essential”) were: nondisruptive to workflow (mean 4.47, SD 0.66; 91%, 95% CI 81%‐96%), positive patient outcomes (mean 4.35, SD 0.81; 88%, 95% CI 77%‐94%), clear recommendations (mean 4.14, SD 0.81; 81%, 95% CI 69%‐89%), alignment with clinical judgment (mean 4.11, SD 0.75; 84%, 95% CI 73%‐92%), training provided (mean 3.91, SD 0.97; 70%, 95% CI 57%‐81%), and intuitive interface design (mean 3.89, SD 0.88; 75%, 95% CI 63%‐85%). Internal consistency for the 6-item implementation scale was acceptable (Cronbach α=0.740).

Respondents could select multiple desired AI applications, so percentages are shares of the 213 total selections. The most desired applications were antibiotic selection (41/213, 19.2%), image interpretation (37/213, 17.4%), ventilator management (30/213, 14.1%), sepsis prediction (28/213, 13.1%), deterioration prediction (26/213, 12.2%), fluid management (24/213, 11.3%), and vasopressor management (18/213, 8.5%). Preferences varied by clinical role: nurses prioritized image interpretation, deterioration prediction, and sepsis prediction, while intensivists preferred antibiotic selection and image interpretation. By department, ICU clinicians most valued antibiotic selection (28/136 ICU selections, 20.6%), while ED clinicians showed more evenly distributed preferences across use cases.

#### Exploratory ICU vs ED Subgroup Comparisons

In exploratory subgroup analysis, prior AI use was more common among ED respondents (10/13, 77%; 95% CI 50%‐92%) than among ICU respondents (12/39, 31%; 95% CI 19%‐46%; *P*=.008). No other main outcome differed significantly between the two departments. For example, combined trust for a stable ward patient was 28/39 (72%; 95% CI 56%‐83%) among ICU respondents and 11/13 (85%; 95% CI 58%‐96%) among ED respondents. Full subgroup statistics with 95% Wilson CIs are provided in Multimedia Appendix 9. The ED subgroup was small, so these comparisons are hypothesis generating only.

### Interview Findings

#### Overview

Semistructured interviews were conducted with 11 participants, including physicians (3), APPs (2), and nurses (6). The thematic analysis for the interviews identified 36 detailed themes from approximately 690 coded segments. To support synthesis and clinical interpretability, these themes were organized into six overarching thematic clusters: (1) trust, transparency, and human control (2) alert design, interface, and usability (3) workflow fit and efficiency (4) data accuracy and algorithm concerns (5) training, rollout, and adoption barriers (6) role of AI in clinical decision making. The six clusters serve as the primary analytic structure. The 36 detailed themes, related subthemes, and representative quotations are provided in Multimedia Appendix 10.

Thematic saturation was considered achieved after the tenth interview, when no new codes or subthemes emerged. Following Malterud et al’s [20] information power framework, our interview sample (n=11) was deemed sufficient given the narrow study aim, specific participant group, strong interview dialogue quality, and application of established theoretical frameworks. A frequency-based analysis of thematic codes confirmed progressive code saturation across the final three interviews. This pattern is consistent with the code saturation thresholds described by Hennink et al [21]. A frequency of thematic discussion is illustrated in Figure 1.

**Figure 1.**
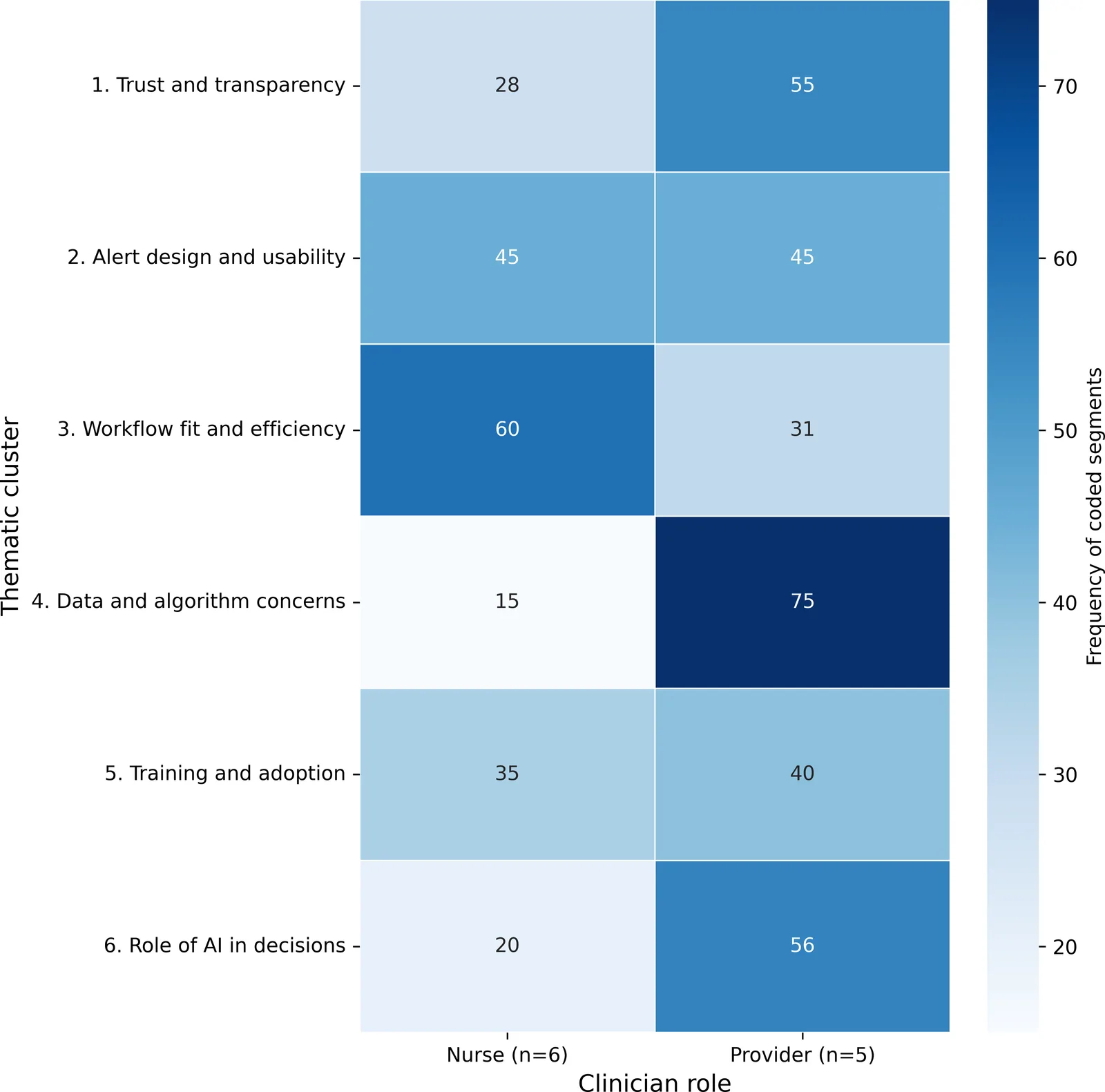
Frequency of thematic discussion by clinician role. This heatmap shows the frequency of coded segments for each thematic cluster, compared between nurses and providers. The “provider” category includes physician, resident, and advanced practice provider participants.

#### Cluster 1: Trust, Transparency, and Human Control

Trust emerged as a central but conditional concept in clinician attitudes toward AI. Participants described trust as dependent on transparency, logic, and the ability to maintain control over the final clinical decision. The need for explainability was repeatedly emphasized, particularly in relation to opaque or “black-box” algorithms: This qualitative finding aligns with survey data showing that 79% (95% CI 67%‐88%) of respondents agreed or strongly agreed that understanding how AI generates its outputs increases trust, while only 47% (95% CI 35%‐60%) agreed that AI systems provide reliable and accurate insights, a 32 percentage-point gap suggesting that trust is contingent on transparency mechanisms rather than assumed reliability.

If it’s going to be a black box in like a neural network, it needs to at least make logical sense.

[P8, Emergency Physician]

Clinicians consistently emphasized the importance of human oversight and override authority:

If you’re thinking of a supportive tool, you should be able to override the tool’s recommendation.

[P9, Nurse Practitioner]

I only trust AI 50%. I still go through the reasoning myself and apply it to the situation at hand.

[P6, Nurse]

Even among those optimistic about AI’s potential, trust was framed as “trust, but verify,” rooted in the belief that decisions must always align with clinical reasoning and patient context.

It depends on the AI system…it’s a trust, but verify. It needs to make logical sense. If it doesn’t, or it doesn’t have robust data behind it, then I’m not gonna trust it.

[P8, Emergency Physician]

AI can pop up and say “Hey, this number looks weird,” but it’s still up to the human to say...we are going to continue with our plan of care...

[P8, Emergency Physician]

Clinicians viewed AI as an “extra set of eyes” that enhances safety but must coexist with human interpretation:

I think it’s an extra set of eyes…an extra layer of safety. But you still need humans putting all the pieces together.

[P5, Nurse]

You never know who put the original information in....If there was some transparency with that, then I think the trust level would be higher.

[P6, Nurse]

#### Cluster 2: Alert Design and Interface Usability

Participants emphasized user control, simplicity, and relevance in the design of alerts and interfaces. The majority preferred opt-in alerts that allow deliberate review rather than automatic notifications. Concise recommendations coupled with links to supporting evidence were considered ideal:

Links to the study rationale as to why it’s doing it are always going to be helpful.

[P8, Emergency Physician]

If I was in clinical settings...I’d love to have a short version, and then click to see the studies when I actually have time.

[P2, Nurse]

Clinicians highlighted the importance of balancing best-practice guidance with individualized patient care:

Version A [opt-in] allows the clinician to take a moment and review which one is better for the patient.

[P2, Nurse]

A subset of participants expressed fatigue toward AI branding, noting that overuse of the term “AI” could reduce acceptance or trigger trust issues:

I’m also feeling this myself, and I do AI research. People are just getting burnt out by the term.

[P11, ICU Physician]

If we see “recommended by AI” on every single thing...then that could trigger trust issues...

[P5, Nurse]

Some envisioned adaptive electronic medical record (EMR) systems that would learn user patterns to reduce redundant tasks and improve efficiency:

The idea of a learning EMR that adapts to my patterns and shows the information I need…without all the extra clicking, would be a real time saver.

[P11, ICU Physician]

#### Cluster 3: Workflow Integration and Efficiency

Several participants viewed AI as a tool to enhance administrative efficiency and relieve documentation burden:

AI impact on nursing workflow…most importantly clinical documentation and note-taking, because many times when you are the ICU clinician, you are busy.

[P6, Nurse]

Ambient listening can save time for providers in terms of summarizing notes.

[P9, Nurse Practitioner]

Clinicians repeatedly noted that workflow integration would determine whether AI-CDSS tools succeed or fail in practice. Systems that disrupt time-sensitive tasks or introduce additional clicks were viewed negatively, even if algorithmically advanced. Reliable data inputs and minimal disruption were essential for trust and adoption:

It’s a great tool, but if you make it harder to use or make me click more, people will avoid it.

[P5, Nurse]

Participants favored AI implementations that fit seamlessly into existing workflows, augmenting rather than complicating clinical processes.

You have to find the right use case. If it’s interrupting the flow or making my job harder, it won’t be used.

[P6, Nurse]

#### Cluster 4: Data Accuracy and Algorithm Concerns

Across interviews, data integrity surfaced as a recurring theme. Participants questioned whether AI models could operate effectively given the variability and inaccuracies of EHR data. Concerns about algorithmic reliability often traced back to inconsistent documentation or sensor readings rather than mistrust of AI itself.

AI is only as good as the data it’s trained on.

[P7, Physician]

There’s a lot of garbage when it comes to vital signs, like a ridiculous amount.

[P8, Emergency Physician]

If you’re using a model that’s based on garbage…it will harm people.

[P1, Nurse]

Clinicians worried that even a well-designed algorithm could amplify inequities or generate unsafe recommendations if trained on biased or incomplete datasets.

It would be harmful in patient populations where we don’t have a lot of data…broadly applying things that may be true for the majority may not apply to the minority.

[P11, ICU Physician]

A related barrier noted by several participants was automation bias, reflecting apprehension that clinicians might overtrust AI outputs without understanding their context or training data:

I worry about automation bias…people treating what AI says as golden, without knowing how it was trained or the context it’s appropriate for.

[P11, ICU Physician]

#### Cluster 5: Training, Rollout, and Adoption Barriers

The theme of training and implementation was strongly represented across participants. Many clinicians cited past experiences with EHR rollouts that lacked context or interactivity:

I’ve had experiences where things in the clinical setting get rolled out without much explanation or any explanation at all.

[P10, Nurse Practitioner]

There was no opportunity to interact or ask questions…I would want to know the reason it was being implemented, the benefit of it.

[P2, Nurse]

Participants consistently favored peer-led “super user” programs that provided accessible, hands-on training:

Emory is very good at doing that, any time a new system is introduced, we have super users.

[P6, Nurse]

I think if there was a dedicated person…that would be helpful; everyone is already overburdened with work.

[P3, Resident]

Most participants rejected static, online formats:

I don’t want to take a class, and I don’t want to do an online course or PowerPoint.

[P3, Resident]

Effective rollout was described as transparent, contextualized, and supported by leadership and time allocation.

#### Cluster 6: Role of AI in Clinical Decision-Making

Clinicians characterized AI as a complementary aid that can enhance clinical awareness and decision-making but not replace human expertise. They underscored that human touch and situational awareness remain irreplaceable. AI was described as a diagnostic “extension,” comparable to tools like ultrasound:

I think it should be an extension of our physical exam…just like ultrasound…but with more caution.

[P11, ICU Physician]

My ideal use is: reinforce what I already thought, or help me think in a new way.

[P8, Emergency Physician]

You can’t replace the hands and body of a nurse.

[P5, Nurse]

Several participants recognized AI’s emerging role in detecting subtle findings and supporting clinical intuition, expressing optimism.

I think it’s going to be a pillar of medical care…for predicting decompensation based on vitals and labs.

[P4]

AI program caught a small subdural hematoma the radiologist missed...it was really cool to see that.

[P9]

We’re still in the infancy for it….But I think this is going to be the way in which we get a better sense of personalized medicine and healthcare.

[P11, ICU Physician]

Despite optimism, clinicians cautioned that overreliance could erode core clinical skills, noting that excessive reliance on automated systems could diminish traditional clinical capabilities:

My clinical exam skills are not on par with my predecessors…the people coming after me, their skill sets have eroded even more.

[P11, ICU Physician]

A comprehensive summary integrating survey and interview findings is presented in Table 2.

**Table 2.** Joint display: integration of survey and interview findings.

Survey finding (quantitative)	Interview theme (qualitative)	Integration and interpretation
Trust decreases with acuity: 75% stable → 47% deteriorating → 44% each for ICU and ED (Cochran Q=30.40, *P*<.001)	Cluster 1: “I only trust AI 50%...I still go through the reasoning myself” (P6). Trust described as conditional and context-dependent.	Convergent: Both data sources confirm acuity-dependent trust. Qualitative data reveals this reflects clinicians’ belief that higher-acuity decisions require greater human judgment, not blanket AI distrust.
55% of AI users cited insufficient training as barrier; 55% cited lack of trust	Cluster 5: “Things get rolled out without much explanation” (P4). Participants favored peer-led “super user” programs over online training.	Expansion: Survey identifies training as top barrier; interviews explain why past negative rollout experiences create lasting skepticism. Training format (hands-on vs online) matters more than training availability.
Exploratory A/B preference observation: 10/11 participants preferred opt-in alerts; 8/11 preferred non-AI-branded alerts	Cluster 2: “People are just getting burnt out by the term [AI]” (P11). Preference for clinician-controlled, evidence-framed alerts.	Convergent: Strong alignment between survey preferences and interview explanations. Debranded, opt-in design reflects desire for autonomy and reduced alert fatigue. These exploratory mock-alert preferences require replication and should not be interpreted as causal design conclusions.
47% agreed AI provides reliable insights; 79% concerned about overreliance	Cluster 4: “AI is only as good as the data it’s trained on” (P7). Data quality concerns central to distrust.	Complementary: Survey captures magnitude of distrust; interviews reveal root cause, concerns about training data quality and EHR data integrity rather than AI capability per se.
100% rated transparency as important or very important; 79% said understanding outputs increases trust	Cluster 1: “If it’s a black box...it needs to at least make logical sense” (P8). Transparency enables “trust, but verify” approach.	Convergent: Universal agreement on transparency importance. Interviews reveal clinicians want rationale not for blind acceptance but for active verification, consistent with maintaining clinical expertise.
54% view AI as “helpful but supplementary”; 68% agree AI complements expertise	Cluster 6: “It should be an extension of our physical exam” (P11). AI as “extra set of eyes,” not replacement.	Convergent: Majority frame AI as augmentation tool. Both sources align on AI-as-supplement, with interviews providing the metaphor of AI as safety net.
No direct survey item on AI branding (emergent finding from interviews only)	Cluster 2: “If we see recommended by AI on every single thing...that could trigger trust issues” (P5).	Emergent: AI-branding fatigue identified exclusively through qualitative data, a novel finding that could not have been captured by survey alone, demonstrating the value of the mixed-methods design.

### A/B Testing

In exploratory A/B testing of mock CDS alerts, 91% (10/11) of participants preferred an opt-in version over an opt-out version. When comparing a CDSS alert with a short, concise recommendation vs a more thorough explanation of the recommendation including a hyperlink to the study evidence, most participants (7/11, 64%) preferred the thorough explanation with the study rationale hyperlink included, while 18% (2/11) preferred having a balance of brief, concise alert messages, with a clickable hyperlink to the study evidence for reference at point of care. Seventy-three percent of participants (8/11) preferred the CDSS alert that did not mention AI in its recommendation. All respondents emphasized the importance of AI explainability (Figure 2). Further discussion of interface design considerations derived from the A/B testing results is presented in Multimedia Appendix 11. Each A/B pair varied several design features at once and the presentation order was fixed, so these percentages are exploratory preference observations. They should not be attributed to any single design element, and they require replication before generalization.

**Figure 2.**
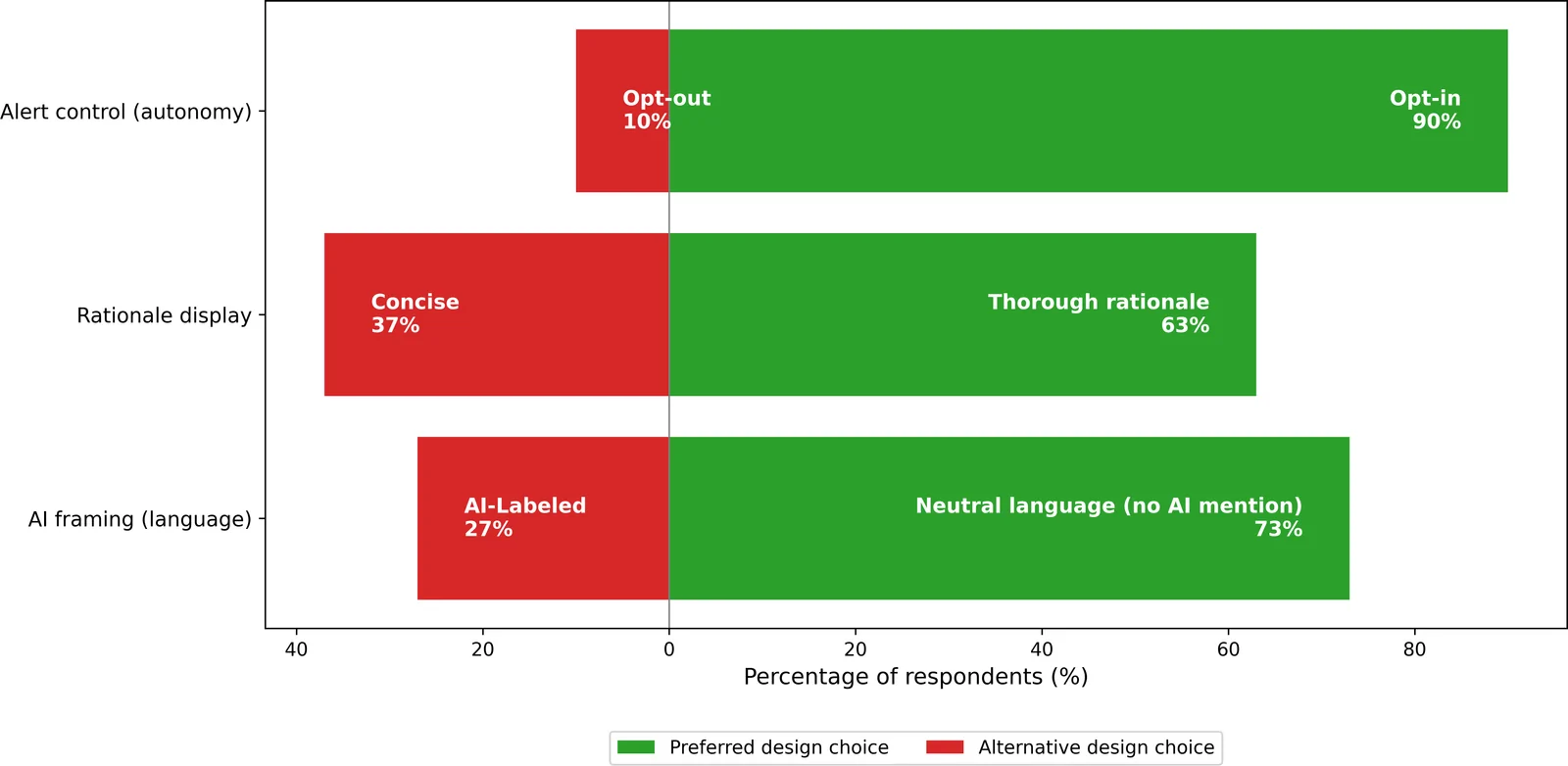
A/B testing preferences.

### Case Scenario

A clinical sepsis scenario was presented to a subgroup of five providers (three physicians and two APPs). Their perceptions of five potential AI-driven interventions revealed a varied pattern (Figure 3). Clinicians were skeptical of AI for straightforward, protocolized tasks like initial fluid resuscitation and antibiotic selection in an uncomplicated case and were more receptive when decisions involved higher complexity like ongoing fluid management, vasopressor selection, and corticosteroid initiation, though this was often conditional on trusting the underlying data. The detailed responses are summarized in Multimedia Appendix 12. This component included only five providers and excluded nurses, although nurses participated in all other survey and interview components. It is presented as illustrative qualitative evidence rather than a basis for broader conclusions.

**Figure 3.**
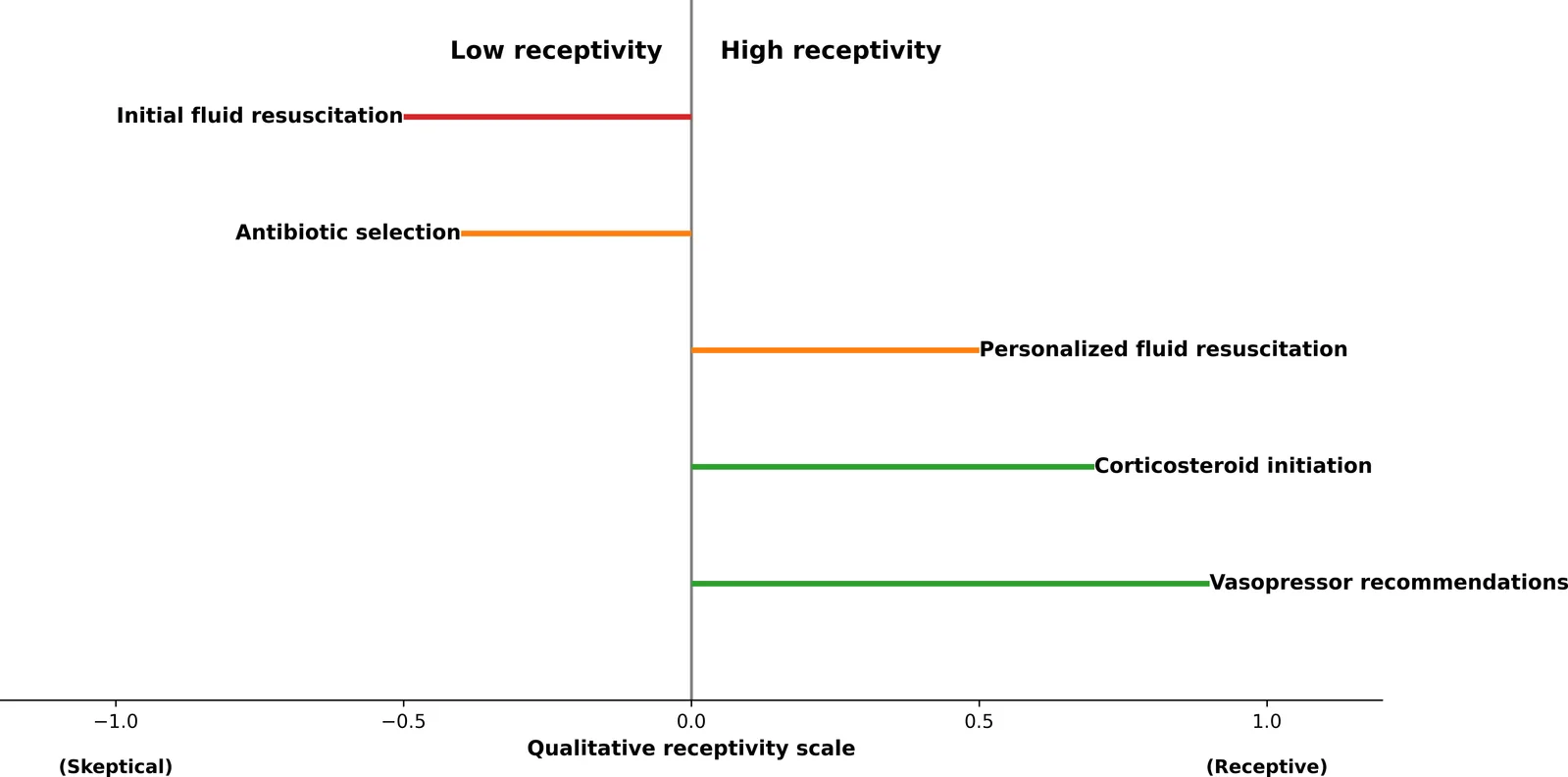
Physicians’ perceptions on AI-driven clinical decision support systems in sepsis case scenarios. We created a qualitative receptivity scale (–1.0=skeptical to +1.0=receptive) to illustrate clinician responses to the five AI-driven interventions. Placement on the scale was assigned by the coding reviewers through consensus, informed by thematic coding of case scenario responses and not derived from a quantitative measure, with left indicating skepticism, center reflecting conditional acceptance, and right showing consistent openness. The scale is conceptual rather than numerical, serving to highlight relative differences in receptivity.

## Discussion

### Principal Findings

This study demonstrates that while clinicians in high-acuity settings are optimistic about AI’s potential, their willingness to adopt these tools is contingent on a core set of principles. The AI-CDSS should be transparent in its algorithm, integrate seamlessly into complex workflows, and respect clinician autonomy and judgment. To bridge the gap between technological innovation and meaningful clinical use, the focus must shift from the capabilities of the algorithm alone to address the needs and perceptions of the clinicians for successful implementation. Our findings reflect perceived acceptability, elicited with mock alerts and hypothetical scenarios, and not actual use, implementation success, or patient outcomes. The study is therefore best understood as an early-stage formative evaluation, consistent with Phase 0 of the Developmental and Exploratory Clinical Investigations of Decision Support Systems Driven by AI (DECIDE-AI) framework [22].

Viewed through the SEIPS 2.0 framework [10], our findings map onto all five work system components: people (clinicians’ trust varies by role, experience, and acuity context), tasks (AI-CDSS must fit complex, time-pressured clinical decision-making), tools and technologies (alert design preferences including opt-in mechanisms and debranded interfaces), organization (training infrastructure, rollout strategy, and peer champion models), and environment (ICU and ED contexts with distinct workflow pressures). Our results demonstrate bidirectional interactions: organizational training deficits reduce trust in the technology, while poor technology design increases task burden, illustrating why isolated interventions targeting single SEIPS components are unlikely to succeed. Interpreted through Hoff and Bashir’s [13] three-layer model of trust, the acuity-dependent trust gradient reflects situational trust, and the AI-branding fatigue described in interviews reflects learned trust shaped by prior technology experiences [8]. While several of these findings converge with prior work, such as clinicians valuing transparency and explainability being important for adoption [23-31], this study identifies three distinctive insights for successful CDSS creation: preference for lower acuity scenarios, a preference-effectiveness paradox, and emphasis on training and implementation. We use the term preference-effectiveness paradox to mean that the alert designs clinicians prefer are not necessarily the designs that prove most effective in practice.

Initial AI-CDSSs should be strategic to target lower acuity scenarios while maintaining clinician autonomy to foster clinician confidence in their implementation. The demonstration of acuity-dependent trust in this study (decreasing from 75% [43/57] in stable patients to 44% [25/57] in critically ill patients) suggests a graduated, SEIPS-informed implementation strategy that accounts for task complexity. We frame this strategy as a recommendation for future testing because this study did not evaluate implementation or measure changes in confidence over time. This strategy allows a CDSS to demonstrate its reliability and value in a safer context, creating a foundation of trust before it is applied to higher acuity situations. Study participants most frequently requested AI-CDSSs for radiology and antibiotic selection, which are both lower acuity scenarios. Adaptability of AI tools at lower stakes situations has been shown to be effective in clinical scenarios like reading chest X-rays (CXRs) [32-34]. While implementation of AI assistance in CXR reading has been directed at radiologists, these applications may also have value in integration for critical care or ED clinicians. For instance, AI has the capability to effectively identify a pneumothorax, which could assist in earlier detection in resource-limited hospitals [35]. This algorithm could then, through a CDSS, prompt the provider to review or re-examine the CXR. For antibiotic selection, an AI-CDSS could make recommendations based on the hospital antibiogram and patient’s former infections and antibiotic exposure. One study showed promising development of an AI algorithm that effectively detected *Stenotrophomonas maltophilia* with antibiotic resistance, and a CDSS could more rapidly alert a clinician compared to the standard practice [36]. Integration of AI in these lower acuity situations could help establish trust between the clinician and AI-CDSS [37]. As exposure to AI-CDSSs increases, these perceptions may change, and ongoing evaluation would be helpful to capture evolving attitudes and possibly allow for higher acuity applications. Our understanding of acuity with AI-CDSS recommendations remains theoretical, and future studies should compare the deployment of AI-CDSSs in variable acuity settings.

Our study demonstrated the importance of preserving clinician autonomy for acceptance of the AI-CDSS, which can be done by designing an “opt-in” over an “opt-out” system. Preserving clinician control over when and how AI recommendations are provided may support trust and perceived usefulness of the system. However, it is important to acknowledge that clinician preferences do not necessarily predict optimal AI-CDSS effectiveness in practice. Further studies are needed to compare different AI-CDSS design models in actual clinical settings. While opt-in designs may be preferred, their passive nature may result in lower compliance rates compared to more intrusive opt-out configurations in practice, as evidenced from prior clinical decision support research [5]. This demonstrates the preference-effectiveness paradox, in which autonomy-preserving designs may be preferred in fast-paced settings like the ED and ICU workflows, but optional alerts may be missed or deprioritized. Autonomy-preserving designs should therefore be paired and evaluated with monitoring of alert uptake and safety outcomes in future research. From a SEIPS perspective, this highlights an important tension between the person (clinician preferences) and organization (system effectiveness) components and underscores the need for future implementation studies to evaluate different alert design strategies in real clinical settings.

Interestingly, there was a strong preference for alerts that did not mention AI. It may reflect AI fatigue, distrust, framing effects, or concern about accountability. Debranding also creates an ethical tension with transparency because clinicians and patients may reasonably expect to know when a recommendation is AI-derived [23,26]. This exploratory finding requires replication and dedicated study before it informs design practice.

Finally, our study highlights that even a perfectly designed tool will fail if not implemented thoughtfully. “Insufficient training” was identified as a primary barrier, along with causing lack of trust. Participants reflected on prior EHR rollouts that were characterized by one-way, module-based training and minimal opportunity for feedback, which often resulted in disengagement. Conversely, interactive, peer-led “super-user” models were described as more effective and engaging. These “super-users” should have a strong understanding of the AI systems and algorithms, as well as familiarity with organizational change management processes. In addition to technical expertise, trainers should possess strong communication and listening skills to effectively engage with clinical teams and translate technical system capabilities into practical clinical use [38]. Consistent with prior studies, participants reported that training is more meaningful when it explains not only how to use the system but also why it is being implemented and what value it provides [39,40]. Participants also highlighted the importance of transparency about AI model development, including its training data and validation process [41,42]. Broader literature similarly points to the significance of addressing “change fatigue” and providing protected time for clinicians to participate in training to ensure effective engagement [43]. Training that is interactive, context-specific, and psychologically informed may mitigate resistance and improve confidence in AI-CDSS use [39,44].

### Limitations and Future Directions

This study should be interpreted within the context of several limitations. First, data collection occurred within a single academic medical center, which may limit generalizability. Perceptions may differ in community hospitals and resource-limited settings with less AI exposure, and a multicenter trial is an explicit next step. Second, both interview and survey participation were voluntary, which may have introduced selection bias toward individuals with particularly strong opinions about AI. Recruitment channels were open-ended, so a response rate could not be computed, and respondents could not be compared with the underlying clinician population. In addition, the positive confidence estimates among respondents with prior AI use should be interpreted cautiously because this subgroup was small (n=22); positive selection of AI-engaged clinicians may therefore bias trust and adoption findings upward. Third, the mock alerts used in A/B testing and clinical scenarios may not fully capture the complexity of real-time clinical decision-making. In addition, these mock alerts were derived from a single AI-CDSS prototype associated with the PRECISE trial, and several members of the research team are also investigators in that trial. Therefore, findings should be interpreted as formative guidance rather than broadly generalizable evidence of AI-CDSS implementation across settings. Fourth, our sample size may have limited the power for some findings. The sample was primarily ICU based; ED findings are exploratory, and several cross-tabulations had sparse cells and are reported as sensitivity analyses. Fifth, trust in AI is dynamic and evolves with exposure [8]; our cross-sectional design captures a single time point. Future work could include a longitudinal extension, such as a resurvey of the same cohort after deployment of the PRECISE trial AI-CDSS, to capture learned trust trajectories [13,22]. Finally, this study focused exclusively on clinician perceptions of AI-CDSSs; however, the growing literature on shared decision-making in AI-augmented care emphasizes that patient perspectives are equally critical for successful AI-CDSS adoption [45]. We also note that SEIPS 3.0 has extended the model to include patient journey considerations [46]. Future directions should include multicenter replication of these findings, longitudinal evaluation of the temporal evolution of clinician trust in AI-CDSSs, and input from patient and public advocates to better understand patient-centered acceptability, transparency, and perceived benefit.

In conclusion, this study demonstrates that adoption of AI-CDSSs in critical care is not solely a technical issue but a human-factors challenge that requires focus on trust, transparency, and workflow compatibility. These conclusions are based on clinician perceptions, mock alerts, and hypothetical scenarios in a single academic medical center; the proposed implementation strategies should be interpreted as exploratory and hypothesis-generating. Their effectiveness and generalizability require validation through prospective multicenter studies and real-world AI-CDSS implementation.

## Supplementary material

10.2196/95472 Multimedia Appendix 1 A/B testing mock-up pair 1 – critical alert design. Version A displays two user-action buttons (“ACCEPT”/“REJECT”) allowing clinicians to confirm or decline a recommended fluid change. Version B shows an auto-executed substitution (“Lactated Ringer’s Infusion has been ordered instead”), requiring manual cancellation to reverse.

10.2196/95472 Multimedia Appendix 2 A/B testing mock-up pair 2 – evidence-framing design. Version A presents a brief alert message without supporting citation. Version B adds an evidence-based rationale and a hyperlink to the referenced research.

10.2196/95472 Multimedia Appendix 3 A/B testing mock-up pair 3 – algorithm-attribution design. Version A explicitly attributes the recommendation to an AI algorithm. Version B conveys the same guidance using clinical logic and omits any mention of AI.

10.2196/95472 Multimedia Appendix 4 A/B testing alert designs presented to interview participants.

10.2196/95472 Multimedia Appendix 5 Clinical case scenario for physicians and advanced practice providers.

10.2196/95472 Multimedia Appendix 6 Survey questionnaire.

10.2196/95472 Multimedia Appendix 7 Interview questionnaire.

10.2196/95472 Multimedia Appendix 8 Supplementary statistical tables.

10.2196/95472 Multimedia Appendix 9 Exploratory intensive care unit (ICU) vs emergency department (ED) subgroup analysis.

10.2196/95472 Multimedia Appendix 10 Comprehensive overview of 36 themes with subthemes and representative quotes.

10.2196/95472 Multimedia Appendix 11 Supplementary insights.

10.2196/95472 Multimedia Appendix 12 Summary of clinician perceptions in the sepsis scenario.

10.2196/95472 Checklist 1 GRAMMS checklist.

10.2196/95472 Checklist 2 CHERRIES checklist.

10.2196/95472 Checklist 3 COREQ checklist.

## Abbreviations

APP: advanced practice provider
CDSS: clinical decision support system
CHERRIES: Checklist for Reporting Results of Internet E-Surveys
COREQ: Consolidated Criteria for Reporting Qualitative Research
CXR: chest X-ray
DECIDE-AI: Developmental and Exploratory Clinical Investigations of Decision Support Systems Driven by AI
ED: emergency department
EHR: electronic health record
EMR: electronic medical record
GRAMMS: Good Reporting of a Mixed Methods Study
ICU: intensive care unit
IRB: institutional review board
PRECISE: Precision Resuscitation With Crystalloids in Sepsis
SEIPS: Systems Engineering Initiative for Patient Safety
TAM: technology acceptance model
UTAUT: unified theory of acceptance and use of technology
