# Supplementary material for: Clinician Trust and Human Factors in AI-Enabled Clinical Decision Support in Acute Care: Mixed Methods Study
Source: JMIR Hum Factors. 2026 Oct 2;13:e95472. doi: 10.2196/95472 (PMC13633065; doi:10.2196/95472)
Supplement: Supplementary file 1 [file humanfactors-v13-e95472-s004.docx]

These illustrate the three sets of mock-up alert designs that were presented to the 11 interview participants to elicit feedback on usability and framing.

Set 1:

- Version A: This design presented an "Alternative Recommended" and provided two clear choices: an "ACCEPT" button to switch to the recommended fluid and a "REJECT" button to continue with the originally selected order.
- Version B: This design informed the user that the system had already acted on their behalf ("Lactated Ringer’s Infusion has been ordered instead."). To disagree, the clinician was instructed to manually "cancel the order and re-order".

Set 2:

- Version A: This alert offered a more concise statement without a direct link to evidence.
- Version B: This alert provided a more thorough, evidence-based rationale, including a hyperlink to supporting research

Set 3:

- Version A: This design directly attributed the recommendation to the algorithm.
- Version B: This design framed the recommendation based on clinical logic and omitted any direct mention of AI.
