# Supplementary material for: Clinician Trust and Human Factors in AI-Enabled Clinical Decision Support in Acute Care: Mixed Methods Study
Source: JMIR Hum Factors. 2026 Oct 2;13:e95472. doi: 10.2196/95472 (PMC13633065; doi:10.2196/95472)
Supplement: Supplementary file 2 [file humanfactors-v13-e95472-s005.docx]

A 54-year-old male with a past medical history of HTN, DM Type 2 on insulin, HLD, CKD stage 3, and obesity presents to the ED with 2 days of fevers, nonproductive cough, fatigue, and had poor oral intake. In the ED he is hypotensive (BP: 90/55, MAP 60), HR 105, T: 39.1, RR 24 and SpO2 91%. He is placed on NC at 2L. Blood cultures and sputum are ordered, and he is started on antibiotics. (Would you want AI to suggest antibiotics?)

He receives 1L fluid bolus and temporarily his MAP improves to > 65. (Would you want AI to suggest the type or quantity of crystalloids for initial resuscitation?)

The nurse comes to get you, and his MAP is now < 60. You do a passive leg raise and his pulse pressure widens significantly. You give an additional 1 L fluid bolus as he is transferred to the ICU. (Would you want AI to provide personalized fluid resuscitation recommendations?)

On arrival to the ICU, the initial MAP is 55 and the patient has increased work of breathing. The patient is started on levophed gtt and titrated up to 0.2 mcg/kg/min (Would you like AI to make vasopressor recommendations or titration goals?)

Vasopressin gtt is initiated due to persistent MAPs in the 60s. (Would you like AI to make recommendations on corticosteroid initiation?)
