# Supplementary material for: Clinician Trust and Human Factors in AI-Enabled Clinical Decision Support in Acute Care: Mixed Methods Study
Source: JMIR Hum Factors. 2026 Oct 2;13:e95472. doi: 10.2196/95472 (PMC13633065; doi:10.2196/95472)
Supplement: Supplementary file 3 [file humanfactors-v13-e95472-s006.docx]

1. What is your current clinical role?
2. How many years have you been practicing in your current role?
3. Where in the hospital do you primarily work in?
4. Have you utilized an Artificial Intelligence (AI) algorithm in your clinical practice?
5. How often do you use AI-enabled Clinical Decision Support Systems?
6. How confident are you in the recommendations provided by AI tools?
7. What barriers have you experienced while using AI tools?
8. To what extent do you agree with the following statement: The AI tools have positively impacted my clinical workflow.
9. What is your perception of AI's role in healthcare?
10. Please indicate your level of agreement with the following statements about AI in healthcare:

a. AI improves the accuracy of clinical decisions.

b. AI systems enhance efficiency in patient care.

c. AI helps reduce variability in clinical decision-making.

d. AI complements clinical expertise effectively.

e. Over-reliance on AI poses risks to patient outcomes.

11. How do you anticipate AI-based tools could impact your clinical workflow?
12. How important is it for an AI tool to display the rationale behind its recommendations?
13. In which clinical areas would you like to use the AI recommendations? (Select all that apply)
14. Which type of AI-driven fluid recommendation would be most useful in your clinical practice? Please rank the following in order of usefulness, with being the most useful and 4 being the least useful. (Determination of amount of initial fluid resuscitation in the ED, Determination of continued fluid resuscitation versus initiating vasopressors, Determination of the type of fluid resuscitation (crystalloids versus colloids, normal saline versus balanced crystalloids), Determination of when to begin de resuscitation and diuresis)
15. In your opinion, what is the biggest advantage and challenge of using AI in clinical practice?
16. Do you have concerns about the ethical implications of AI in clinical decision-making?
17. How would you trust AI recommendations in the following situations: (Stable patient on the wards, deteriorating patient on the wards, critically-ill patient in the ICU, Undifferentiated patient in the ED)
18. Indicate your level of agreement with the following statements about trust in AI:

a. AI systems provide reliable and accurate insights

b. Understanding how AI generates its outputs increases my trust in its recommendations.

c. AI systems offer insights that complement clinical expertise

19. How important are the following factors in making an AI system effective for your use? (Clear recommendations, Intuitive interface design, Alignment with clinical judgment, Training provided, Positive impact on patient outcomes, Non-disruptive to the clinical workflow)
