# Supplementary material for: Clinician Trust and Human Factors in AI-Enabled Clinical Decision Support in Acute Care: Mixed Methods Study
Source: JMIR Hum Factors. 2026 Oct 2;13:e95472. doi: 10.2196/95472 (PMC13633065; doi:10.2196/95472)
Supplement: Supplementary file 4 [file humanfactors-v13-e95472-s007.docx]

1. Can you tell me about your current clinical role and experience managing critically ill patients?
2. What is your experience with clinical decision support systems? What is your experience using AI-driven CDSS?
3. What are your general thoughts on AI-driven clinical decision support systems/ AI in decision-making?
4. Where do you think AI would be most helpful in clinical practice?
5. What concerns do you have about AI in clinical care?
6. Do you think AI should guide clinical decisions, or simply provide information while leaving the decision entirely to the clinician?
7. What do you think of AI’s role in the following areas, would you use it? Out of these, which category do you think AI would be the most helpful? Can you rank top 2? Why? Other than those present in the list, is there any other category or department where you think AI would be useful?
8. Are there any areas where you think AI would be inferior or harmful? Why?
9. What do you think about the use of AI in ED/ICU? Do you think it is needed and will be helpful? How do you think it will affect your work and do you have any concerns regarding its implementation?
10. Do you think there are any barriers to implementing AI in ICU/ED?
11. In what ways do you currently recognize when a patient is deteriorating and needs escalation of care?
12. Do you think AI could help in recognizing when a patient is deteriorating and needs escalation in ED/ICU? Why? Why not?
13. What specific tasks in your daily nursing workflow do you think AI could support?
14. How much do you trust an AI system’s recommendation?
15. What factors would make you more or less likely to trust an AI recommendation?
16. Would seeing a confidence score or rationale from the study (e.g., link to the literature) make you more likely to trust or follow an AI recommendation? Why or why not?
17. How would you want to be trained on AI tools in clinical practice? When an AI system is introduced, what would be the best way to educate and communicate its use to you?
18. How do you see AI evolving in clinical practice, and what are your hopes or concerns for its future?
19. A/B Testing specific: Between these two versions, which one would you find helpful? And why? Which one do you find clearer and more actionable? Why? Between the two versions (opt-in vs opt-out), which version is better to integrate into CDSS? Why? What additional features would improve the AI alert? If you could design an ideal AI alert for your workflow, what features would it include or exclude?
