# Supplementary material for: Clinician Trust and Human Factors in AI-Enabled Clinical Decision Support in Acute Care: Mixed Methods Study
Source: JMIR Hum Factors. 2026 Oct 2;13:e95472. doi: 10.2196/95472 (PMC13633065; doi:10.2196/95472)
Supplement: Supplementary file 5 [file humanfactors-v13-e95472-s008.docx]

Table S3. Medians and IQRs for all Likert-type items (N=57), reported alongside the means and SDs given in the main text. Agreement and trust items use 5-point scales from 1 (Strongly Disagree / Strongly Distrust) to 5 (Strongly Agree / Strongly Trust); implementation items use 1 (Not important) to 5 (Essential).

| **Item** | **Mean (SD)** | **Median (IQR)** |
| --- | --- | --- |
| AI improves accuracy of decisions | 3.54 (0.78) | 4 (3-4) |
| AI enhances care efficiency | 3.79 (0.80) | 4 (3-4) |
| AI reduces decision variability | 3.51 (0.78) | 4 (3-4) |
| AI complements clinical expertise | 3.74 (0.77) | 4 (3-4) |
| AI insights complement expertise | 3.86 (0.74) | 4 (4-4) |
| AI provides reliable insights | 3.35 (0.88) | 3 (3-4) |
| Over-reliance on AI poses risks | 4.05 (0.91) | 4 (4-5) |
| Understanding AI outputs increases trust | 4.14 (0.97) | 4 (4-5) |
| Trust: stable patient (ward) | 3.88 (0.78) | 4 (4-4) |
| Trust: deteriorating patient (ward) | 3.21 (1.06) | 3 (2-4) |
| Trust: critically ill patient (ICU) | 3.09 (1.12) | 3 (2-4) |
| Trust: undifferentiated patient (ED) | 3.19 (1.04) | 3 (3-4) |
| Importance: non-disruptive to workflow | 4.47 (0.66) | 5 (4-5) |
| Importance: positive patient outcomes | 4.35 (0.81) | 5 (4-5) |
| Importance: clear recommendations | 4.14 (0.81) | 4 (4-5) |
| Importance: alignment with clinical judgment | 4.11 (0.75) | 4 (4-5) |
| Importance: training provided | 3.91 (0.97) | 4 (3-5) |
| Importance: intuitive interface design | 3.89 (0.88) | 4 (4-4) |

Table S4. Pairwise exact McNemar tests for combined trust across patient acuity scenarios, with Bonferroni, Holm step-down, and Benjamini-Hochberg false discovery rate adjustment. Discordant pairs are respondents trusting in the first scenario only / second scenario only. Conclusions are identical under all three procedures.

| **Comparison** | **Discordant pairs** | **Raw exact** | **Bonferroni** | **Holm** | **FDR (BH)** |
| --- | --- | --- | --- | --- | --- |
| Stable patient (ward) vs Deteriorating patient (ward) | 18/2 | *P*<.001 | *P*=.002 | *P*=.002 | *P<*.001 |
| Stable patient (ward) vs Critically ill patient (ICU) | 20/2 | *P*<.001 | *P*<.001 | *P*<.001 | *P*<.001 |
| Stable patient (ward) vs Undifferentiated patient (ED) | 21/3 | *P*<.001 | *P*=.002 | *P*=.001 | *P*<.001 |
| Deteriorating patient (ward) vs Critically ill patient (ICU) | 2/0 | *P*=.50 | *P*=1.00 | *P*=1.00 | *P*=.75 |
| Deteriorating patient (ward) vs Undifferentiated patient (ED) | 6/4 | *P*=.75 | *P*=1.00 | *P*=1.00 | *P*=.90 |
| Critically ill patient (ICU) vs Undifferentiated patient (ED) | 6/6 | *P*=1.00 | *P*=1.00 | *P*=1.00 | *P*=1.00 |

Non-parametric sensitivity analysis on the full 5-point trust ratings: Friedman chi-square=33.5, df=3, *P*<.001; Kendall W=0.20. This confirms the Cochran Q result without dichotomizing the trust scale.

Table S5. Internal consistency of multi-item scales: Cronbach alpha and McDonald omega (total, from a one-factor model on the Pearson correlation matrix). The AI Perception scale comprises the accuracy, efficiency, variability, both complementarity, and reliability items; the negatively worded over-reliance item is analyzed as a standalone item.

| **Scale** | **Cronbach alpha** | **McDonald omega** |
| --- | --- | --- |
| AI Perception (6 items) | 0.891 | 0.895 |
| Trust Beliefs (3 items) | 0.743 | 0.782 |
| Implementation Factors (6 items) | 0.740 | 0.746 |

Table S6. Sensitivity analyses for sparse cross-tabulations. Because more than 20% of expected cell counts are below 5, the asymptotic chi-square approximation is unreliable and these results are exploratory. Monte Carlo P values are from 100,000 simulated tables with fixed margins (seed 20260706).

| **Association** | **Chi-square** | **df** | **Asymptotic** | **Monte Carlo** | **Cramér V** | **Cells with expected count <5** |
| --- | --- | --- | --- | --- | --- | --- |
| Department (4 levels) x AI perception | 17.45 | 9 | *P*=.04 | *P*=.08 | 0.32 | 75% |
| Clinical role (8 levels) x prior AI use | 16.51 | 7 | *P*=.02 | *P*=.01 | 0.54 | 75% |
