# Supplementary material for: Clinician Trust and Human Factors in AI-Enabled Clinical Decision Support in Acute Care: Mixed Methods Study
Source: JMIR Hum Factors. 2026 Oct 2;13:e95472. doi: 10.2196/95472 (PMC13633065; doi:10.2196/95472)
Supplement: Supplementary file 6 [file humanfactors-v13-e95472-s009.docx]

Main survey outcomes compared between ICU respondents (n=39) and ED respondents (n=13). Respondents from other settings (n=5) are excluded. Proportions are reported as n/N (%) with 95% Wilson score CIs. P values are from Fisher exact tests (binary outcomes, Table S1) and Mann-Whitney U tests (ordinal outcomes, Table S2). These comparisons are exploratory and hypothesis generating: the ED subgroup is small, no adjustment is made for multiple comparisons, and absence of evidence should not be read as evidence of equivalence.

Table S1. Binary outcomes by department (n/N, %, 95% Wilson CI).

| **Outcome** | **ICU (n=39)** | **ED (n=13)** | **Fisher exact** |
| --- | --- | --- | --- |
| Prior AI use in clinical practice | 12/39 (31%; 19-46) | 10/13 (77%; 50-92) | *P*=.008 |
| Trust: stable patient (ward) | 28/39 (72%; 56-83) | 11/13 (85%; 58-96) | *P*=.48 |
| Trust: deteriorating patient (ward) | 17/39 (44%; 29-59) | 8/13 (62%; 36-82) | *P*=.34 |
| Trust: critically ill patient (ICU) | 16/39 (41%; 27-57) | 7/13 (54%; 29-77) | *P*=.52 |
| Trust: undifferentiated patient (ED) | 14/39 (36%; 23-52) | 8/13 (62%; 36-82) | *P*=.12 |
| Perceives AI as helpful or transformative | 30/39 (77%; 62-87) | 11/13 (85%; 58-96) | *P*=.71 |
| Ethical concerns about AI | 26/39 (67%; 51-79) | 10/13 (77%; 50-92) | *P*=.73 |
| Rates transparency "very important" | 29/39 (74%; 59-85) | 10/13 (77%; 50-92) | *P*=1.00 |
| Agrees over-reliance poses risks | 30/39 (77%; 62-87) | 12/13 (92%; 67-99) | *P*=.42 |
| Agrees understanding outputs increases trust | 28/39 (72%; 56-83) | 12/13 (92%; 67-99) | *P*=.25 |
| Agrees AI provides reliable insights | 17/39 (44%; 29-59) | 7/13 (54%; 29-77) | *P*=.54 |

Table S2. Ordinal outcomes by department, median (IQR) on 5-point scales (trust: 1=Strongly Distrust to 5=Strongly Trust; agreement: 1=Strongly Disagree to 5=Strongly Agree).

| **Outcome** | **ICU median (IQR)** | **ED median (IQR)** | **Mann-Whitney U** |
| --- | --- | --- | --- |
| Stable patient (ward) | 4 (3-4) | 4 (4-5) | *P*=.19 |
| Deteriorating patient (ward) | 3 (3-4) | 4 (3-4) | *P*=.38 |
| Critically ill patient (ICU) | 3 (2-4) | 4 (3-4) | *P*=.63 |
| Undifferentiated patient (ED) | 3 (3-4) | 4 (3-4) | *P*=.19 |
| AI improves accuracy of decisions | 4 (3-4) | 4 (3-4) | *P*=.78 |
| AI enhances care efficiency | 4 (3-4) | 4 (3-4) | *P*=.53 |
| AI provides reliable insights | 3 (3-4) | 4 (3-4) | *P*=.45 |
| Over-reliance on AI poses risks | 4 (4-5) | 5 (4-5) | *P*=.07 |
