# Supplementary material for: Clinician Trust and Human Factors in AI-Enabled Clinical Decision Support in Acute Care: Mixed Methods Study
Source: JMIR Hum Factors. 2026 Oct 2;13:e95472. doi: 10.2196/95472 (PMC13633065; doi:10.2196/95472)
Supplement: Supplementary file 7 [file humanfactors-v13-e95472-s010.docx]

| **Theme** | **Subtheme** | **Clinician Quotes** |
| --- | --- | --- |
| 1. Administrative Efficiency | Clinical Documentation Support | "My nurses will love AI, because we can now dictate our note..." (P6) |
|  | Workflow Optimization | "Ambient listening... can save time for providers in terms of summarizing notes." (P2) |
|  |  | "We've played with some AI tools for not just bed management, but patient throughput as well..." (P9) |
|  |  | "There are task lists and other things... that prompt you to do the right thing at the right time. AI will have similar benefits..." (P9) |
|  | Time & Efficiency Gains | "AI could be helpful in scheduling appointments... even creating a task list for actionable items from meetings." (P2) |
|  |  | "...a caution would be more buttons to click... but if AI helps catch trends, like vitals dropping and alerts the nurse, that would be helpful." (P5) |
| 2. AI in Healthcare | Perceived Clinical Potential | "We're still in the infancy for it... But I think this is going to be the way in which we get a better sense of personalized medicine and healthcare." (P11) |
|  | Contextual Use in Critical Care | "It's just another tool, like point-of-care ultrasound... It helps reinforce what I'm already thinking." (P11) |
|  | Cautious and Conditional Trust | "It could be useful, but... it requires a clinician's expertise to tease out what is significant clinically and what is not." (P6) |
|  | Explainability and Transparency | "If everything I'm seeing tells me one thing, and the AI says another, I need to know: why is it telling me that?" (P10) |
|  | Concern Over Terminology and Bias | "People are getting AI'd out... maybe don't highlight that it's AI on every single alert." (P11) |
|  | Human Oversight is Essential | "The AI doesn't see the patient. I see the patient. That matters." (P3) |
| 3. Alert Preferences & Design | Preference for Opt-in Design | "Version A allows the clinician to take a moment and review which one is better for the patient... it eliminates the extra work." (P4) |
|  | Preference for Simplicity | "Version A is quick and easy... Version B is too wordy and not necessary. People will skip over it." (P10) |
|  | Need for Rationale | "I love that there's a link to the study rationale... We don't have to go looking for it." (P2) |
|  | Cognitive Overload Avoidance | "Version B doesn't even give me the option. You have to go back, cancel the order it's too much. Version A just highlights it better... less intimidating." (P2) |
| 4. Barriers to Adoption | Data Accuracy Concerns | "I don't trust the vitals... there's too much garbage in. So the algorithm is not going to be successful if it's based on inaccurate data." (P8) |
|  | Transparency & Explainability | "Where did the information come from? Was it the top 10 physicians in fluid management or some wackadoodle online source? That's what makes people trust less." (P6) |
|  | Automation Bias | "I worry about automation bias... people treating what AI says as golden, without knowing how it was trained or the context it's appropriate for." (P11) |
|  | Skill Erosion | "My clinical exam skills are not on par with my seniors. And people coming after me, those skills have eroded even more." (P11) |
| 5. Trust & Adoption | Partial/Conditional Trust | "Only 50% is all I am taking what AI is telling me... I don't just fully trust..." (P1) |
|  | Trust Based on Reliability | "If I had 9 out of 0 times I didn't have to change the generated reply... that would increase my trust." (P8) |
|  | Distrust from Missed Context | "The AI doesn't see the whole picture. Sure, it's 90% confident, but if you have more data, you might realize the patient doesn't have sepsis." (P11) |
| 6. Human-AI Collaboration | AI as Assistant, Not Replacement | "I love the idea of not complete AI... It should be a backup, not the only eye on care..." (P4) |
|  | Shared Decision-Making | "I think it should involve almost everyone... the patient, family, nurses, providers. AI can help with that but it can't be the sole decision-maker." (P5) |
|  | Clinician Autonomy is Critical | "I think you should be able to override the tool's recommendation..." (P10) |
|  | Safety Net Role of AI | "It could catch what a human missed... if a computer can re-skim and say hey, this looks weird..." (P4) |
| 7. Workflow Integration | Burden from Extra Steps | "It's a great tool, but if you make it harder to use or make me click more, people will avoid it." (P5) |
|  | Need for Seamless Fit | "You have to find the right use case. If it's interrupting the flow or making my job harder, it won't be used." (P6) |
| 8. Usability & Interface Preferences | Preference for Clear Layout | "Version A would be more user friendly because of the color and the way it's broken up..." (P2) |
|  | Prescriptive Tone is a Turnoff | "Version B is more prescriptive. I prefer Version A, it's more in line with a supportive tool..." (P10) |
|  | Click Fatigue Frustration | "…there’s a lot of clicking fatigue where I just like click through things to get things done, which I would quickly click through version B, and then be annoyed when I had to reorder something… " (P3) |
| 9. Perceived Clinical Impact | Early Detection & Prediction | "I think it's going to be a pillar of medical care... for predicting decompensation based on vitals and labs." (P4) |
|  | Confidence Boost & Validation | "My ideal use is: reinforce what I already thought, or help me think in a new way." (P11) |
| 10. Risk, Safety & Reliability Concerns | Automation Bias & Blind Trust | "When people take what it says as golden... I think there's a lot of danger in that." (P11) |
|  | Risk of Deskilling | "It could decrease critical thinking in clinicians." (P2)  “I hope that we don't get to a point where all of assessment is based on a computer and not and we lose the human skills (P4) |
| 11. Data Quality & Input Limitations | Garbage In, Garbage Out | "If you're using a model that's based on garbage... it will harm people." (P1) |
|  | Bias in Training Data | "If it has biased data or it's not externally valid... I'm not going to trust it." (P11) |
| 12. Explainability and Model Transparency | Black Box Concerns | "If it's going to be a black box... it needs to at least make logical sense." (P11) |
|  | Desire for Evidence & Rationale | "The rationale... that's what makes you pass. Version B gives you what everyone is looking for..." (P2) |
| 13. Trust Dynamics and Variability | High Personal Trust | "If you ask me, which way do I go? I'm more into trusting AI than not... Trust AI would be 99 plus percent." (P9) |
|  | Contextual Trust | "Generally, I would trust the recommendation, but through a lens..." (P7) |
| 14. Overreliance and Deskilling Concerns | Loss of Clinical Intuition | "It's not all algorithmic thinking. There's an art and an intuition that we haven't been able to replicate." (P11) |
|  | Concern About Learners | "I want learners to understand the rationale... AI is a tool, not a replacement for medical decision-making." (P6) |
| 15. Human Oversight and Final Judgment | Support, Not Supplant | "I think AI starts getting out of hand when it takes that humanness out of medicine..." (P5) |
|  | Final Say Rests with Clinician | "AI can pop up and say 'Hey, this number looks weird,' but it's still up to the human to say... we are going to continue with our plan of care..." (P8) |
| 16. Workflow Disruption and Integration Challenges | Screen Time vs. Patient Time | "We traded [paper records] for spending 55 to 60% of our time staring at a screen instead of taking care of a patient." (P11) |
|  | Unrealized Efficiency Promises | "All these promises of us getting time saved... what if that time is now traded for some other even more time-consuming tasks with AI?" (P7) |
| 17. Training Needs and User Readiness | Need for Dedicated Time | "Inadequate training will make clinicians disinterested in using AI..." (P10) |
|  | Super User Support | "You have to have super users... as a resource person to do orientation." (P6) |
|  | Lack of interaction | "When we switched to Epic…all that was just done on a computer, and there was no opportunity to interact or ask questions. It was a little bit frustrating…we get so many PowerPoints sent to us, but people just click through those screens and don’t really absorb it." (P2) |
| 18. Perceived Utility and Optimism | Future-Oriented Mindset | "AI is the best. The future... AI will be more reliable than anything else." (P6) |
|  | Clinical Impact Realized | "AI program caught a small subdural hematoma the radiologist missed... it was really cool to see that." (P9) |
| 19. Trust Spectrum - Confidence, Caution, and Conditions | High Trust and Faith | "I trust AI 99%... it's a compounded, logically combined information." (P9) |
|  | Conditional Trust | "I only trust AI 50%. I still go through the reasoning myself and apply it to the situation at hand." (P6) |
|  | Cautious Evaluation | "I'd say 7 out of 0. I trust it, but through a lens... similar to getting handoff from a colleague you verify." (P11) |
| 20. Transparency and Explainability | Desire for Source Attribution | "You never know who put the original information in... If there was some transparency with that, then I think the trust level would be higher." (P6) |
|  | Need for Scientific Backing | "Yeah, like a link to the literature behind that particular treatment... I'd want to know what data... is giving it this decision." (P2) |
| 21. Alert Fatigue and Notification Overload | Overwhelming Number of Alerts | "Guess what, when the alarm is too much... We have erratic alarm. But AI is now able to be programmed to prioritize..." (P7) |
| 22. Customization and Opt-In Preferences | Opt-In Seen as Respectful | "Version A... eliminates the extra work and allows the clinician to take a moment and review..." (P4) |
|  | Clinician Autonomy Matters | "You should be able to override the tool's recommendation... it's a supportive tool, not prescriptive." (P2) |
| 23. Simplicity and Cognitive Ease | Avoiding Text Overload | "Visually, it's more appealing... there's just way too many words that I don't want to read..." (P1) |
|  | Simplicity in Emergency Contexts | "If I'm in the clinical setting, I'm running around... I'm counting every second... That's why I like the shorter one." (P8) |
| 24. Trust Through Peer Framing and Human Language | Skepticism Toward AI Labels | "If we see 'recommended by AI' on every single thing... then that could trigger trust issues..." (P5) |
| 25. Desire for Rationale and Evidence Transparency | Need to Know the "Why" | "The more important thing to me as the clinician is, is this evidence-based or not? How did we arrive at this suggestion?" (P10) |
|  | Evidence Inspires Confidence | "I would need to know the paper it was based on. Like the data behind it. You need to know the data before changing practice."(P4) |
| 26. Clinical Judgment Still Reigns | Al as Supplementary, Not Primary | "I think it should be an extension of our physical exam... just like ultrasound... but with more caution." (P11) |
|  | Warnings About Blind Trust in AI | "There's a danger in taking what it says as golden... people may not fully appreciate its pitfalls..." (P11) |
| 27. Alert Relevance and Timing | Misaligned Alert Timing | "...you're juggling many different things. Another source of input needs to be useful, not add to the workload." (P8) |
|  | Burden of Irrelevant Alerts | "Sure, if you're looking at a limited picture, yeah, 90% confidence... but then you realize, oh, the patient actually doesn't have sepsis." (P5) |
| 28. Avoiding Overload - Simplicity and Conciseness | Preference for Short Alerts | "Normally, when I'm making point-of-care decisions, I don't want to read a paragraph." (P8) |
|  | Information Layering Preferred | "If I was in clinical settings... I'd love to have a short version, and then click to see the studies when I actually have time." (P2) |
| 29. Bias, Fairness, and Validity of AI Recommendations | Bias in Data = Bias in Output | "If it has biased data, it's not externally valid... something like that, I'm not going to trust it." (P3) |
|  | Diversity of Training Data Matters | "What was the model trained on? Is this the appropriate model for this patient population?" (P11) |
| 30. Concerns About Explainability and the Black Box | Black Box = No Trust | "If it's going to be a black box... it needs to at least make logical sense. If it doesn't... then I'm not gonna trust it." (P11) |
|  | Desire for Data Source Clarity | "If there was some transparency with that... where did the information come from? ...was it from actual physicians?" (P4) |
| 31. Autonomy, Override, and Final Clinical Judgment | Need for Override Option | "I hope there will be a rule-over button... where, if the provider decides, then the provider can overrule. AI shouldn't be 100%." (P9) |
|  | AI is Just a Recommendation | "It's a recommendation, so you're still having some input as well... and there's always an ability to override it." (P2) |
| 32. Data Quality and "Garbage In-Garbage Out" Concern | Garbage In = Garbage Out | "My concern is garbage in garbage out... if we're gonna do this, we need to be better about inputting data." (P11) |
| 33. Communication, Clinical Handoff, and AI Integration | Communication Gaps in Handoff | "One of the problems is... communication. Handoff is unclear. How much the patient has received..." (P7) |
|  | AI Prompting for Team Alignment | "The nursing staff is so busy... the provider doesn't know the patient has rapidly required that much vasopressor. But Epic knows... if it prompted the provider, that would be helpful." (P2) |
| 34. Documentation Burden and Perceived AI Benefits | Note-Taking Fatigue | "The documentation thing is another big one for me... that would be phenomenal."(P2) |
|  | Deferred Notes Lose Clinical Detail | "Many a time the nurses would defer the documentation... in the interim they might have lost the content. But with AI... they can dictate..." (P1) |
| 35. Equity Concerns and Algorithmic Bias | Data Bias and Representation | "It would be harmful in patient populations where we don't have a lot of data... broadly applying things that may be true for the majority may not apply to the minority." (P11) |
|  | Health Equity and Misuse | "My concern is garbage in garbage out, like health equity regarding all of that... people misusing it..." (P1) |
| 36. Explainability, Confidence Scores, and Transparency | Desire for Source Transparency | "If there was some transparency with that... where did this come from... that kind of thing, I think would help with the trust, for sure." (P6) |
|  | Caution Around Confidence Scores | "I know a person who's 99% confident the earth is flat... I want to know where the information comes from, more than that they are confident." (P11) |
