# Supplementary material for: Clinician Trust and Human Factors in AI-Enabled Clinical Decision Support in Acute Care: Mixed Methods Study
Source: JMIR Hum Factors. 2026 Oct 2;13:e95472. doi: 10.2196/95472 (PMC13633065; doi:10.2196/95472)
Supplement: Supplementary file 8 [file humanfactors-v13-e95472-s011.docx]

Several interface-related factors may further shape clinician engagement with AI-CDSS. Participants frequently expressed a preference for alerts that did not explicitly highlight the AI label, suggesting that subtle interface presentation may influence clinician acceptance. From a SEIPS perspective, this observation reinforces the importance of thoughtful design within the Tools component of the work system and represents a design consideration that has not been prominently documented in prior literature [1]. Participants also consistently emphasized the importance of providing a rationale for AI-generated recommendations, consistent with prior studies demonstrating that transparency and explainability are critical for building trust in AI systems [2-9]. However, comparative studies examining AI-CDSS with versus without accessible algorithm explanations have shown that while explanations are desired, they are rarely viewed in routine practice [10]. These variations on CDSS design need to be evaluated in future studies. Recent work on confidence calibration further supports this perspective: Yu et al. (2025) demonstrated that high-confidence AI predictions (90-99%) were overridden only 1.7% of the time, while minimal transparency diagnoses had a 73.9% override rate, suggesting that dynamic confidence calibration may be equally important as static explainability for building clinician trust in AI-CDSS [11]. These findings indicate that explainability features must balance transparency and clinician autonomy with workflow efficiency, for example through concise explanations or embedded hyperlinks that allow clinicians to access additional information when needed.

References

[1] Stewart J, Freeman S, Eroglu E, et al. Attitudes towards artificial intelligence in emergency medicine. Emerg Med Australas. 2024;36(2):252-265. doi:10.1111/1742-6723.14345 PMID:38044755

[2] Rosenbacke R, Melhus Å, McKee M, Stuckler D. How explainable artificial intelligence can increase or decrease clinicians' trust in AI applications in health care: systematic review. JMIR AI. 2024;3:e53207. doi:10.2196/53207 PMID:39476365

[3] Sibbald M, Abdulla B, Keuhl A, Norman G, Monteiro S, Sherbino J. Electronic diagnostic support in emergency physician triage: qualitative study with thematic analysis of interviews. JMIR Hum Factors. 2022;9(3):e39234. doi:10.2196/39234 PMID:36178728

[4] Bergquist M, Rolandsson B, Gryska E, et al. Trust and stakeholder perspectives on the implementation of AI tools in clinical radiology. Eur Radiol. 2024;34(1):338-347. doi:10.1007/s00330-023-09967-5 PMID:37505245

[5] Amann J, Blasimme A, Vayena E, Frey D, Madai VI; Precise4Q consortium. Explainability for artificial intelligence in healthcare: a multidisciplinary perspective. BMC Med Inform Decis Mak. 2020;20(1):310. doi:10.1186/s12911-020-01332-6 PMID:33256715

[6] Cai CJ, Winter S, Steiner D, et al. “Hello AI”: uncovering the onboarding needs of medical practitioners for human-AI collaborative decision-making. Proc ACM Hum-Comput Interact 2019;3(CSCW):1–24. doi:10.1145/3359206

[7] Tanaka M, Matsumura S, Bito S. Roles and competencies of doctors in artificial intelligence implementation: qualitative analysis through physician interviews. JMIR Form Res. 2023;7:e46020. doi:10.2196/46020 PMID:37200074

[8] Wu Y, Wu M, Wang C, Lin J, Liu J, Liu S. Evaluating the prevalence of burnout among health care professionals related to electronic health record use: systematic review and meta-analysis. JMIR Med Inform. 2024;12:e54811. doi:10.2196/54811 PMID:38865188

[9] Trinkley KE, Kahn MG, Bennett TD, Glasgow RE, Haugen H, Kao DP, Kroehl ME, Lin CT, Malone DC, Matlock DD. Integrating the Practical Robust Implementation and Sustainability Model with best practices in clinical decision support design: implementation science approach. J Med Internet Res. 2020;22(10):e19676. doi:10.2196/19676 PMID:33118943

[10] Bolton WJ, Wilson R, Gilchrist M, et al. The impact of artificial intelligence-driven decision support on uncertain antimicrobial prescribing: a randomised, multimethod study. The Lancet Digital Health. 2025;7(11):100912. doi:10.1016/j.landig.2025.100912

[11] Yu Y, Gomez-Cabello CA, Haider SA, et al. Enhancing clinician trust in AI diagnostics: a dynamic framework for confidence calibration and transparency. Diagnostics (Basel). 2025;15(17):2204. doi:10.3390/diagnostics15172204 PMID:40941691
