# Supplementary material for: Clinician Trust and Human Factors in AI-Enabled Clinical Decision Support in Acute Care: Mixed Methods Study
Source: JMIR Hum Factors. 2026 Oct 2;13:e95472. doi: 10.2196/95472 (PMC13633065; doi:10.2196/95472)
Supplement: Supplementary file 9 [file humanfactors-v13-e95472-s012.docx]

| **AI Intervention** | **Summary of Clinician Perceptions** | **Representative Quote** |
| --- | --- | --- |
| Antibiotic Selection | Conditionally Skeptical. Participants rejected AI for this uncomplicated case but were open to it as a "second check" or for more complex patients with resistance histories. | "No, because I know what I would put him on... he is not too medically complex." (P3) |
| Initial Fluid Resuscitation | Skeptical. Clinicians viewed fluid type as a straightforward decision. The main value was seen in using AI to track fluid quantity to prevent overload. | "...if it prompted me how much fluid the patient has already received, so that I'm not using too much." (P3) |
| Personalized Fluid Resuscitation | Mixed. While most were receptive in principle, this was countered by a significant lack of trust in the quality of real-time EHR data. | "So like I said, garbage in garbage out. I don't know if I I trust that enough." (P8) |
| Vasopressor Recommendations | Generally Receptive. All participants were positive, viewing AI as a helpful prompt for timely, evidence-based care. | "So yeah, I think I think that's a good prompt, because I think sometimes... the provider doesn't know that the patient has rapidly required that much vasopressor." (P3) |
| Corticosteroid Initiation | Conditionally Positive. All respondents were receptive, but acceptance was conditionally tied to trusting the quality and validity of the algorithm's training data. | "As much as I trust the data that it was trusted on." (P11) |
