# Supplementary material for: Clinician Trust and Human Factors in AI-Enabled Clinical Decision Support in Acute Care: Mixed Methods Study
Source: JMIR Hum Factors. 2026 Oct 2;13:e95472. doi: 10.2196/95472 (PMC13633065; doi:10.2196/95472)
Supplement: Supplementary file 10 [file humanfactors-v13-e95472-s013.docx]

Framework: O'Cathain A, Murphy E, Nicholl J. J Health Serv Res Policy. 2008;13(2):92-98.

| **GRAMMS item** | **Where addressed in the manuscript / response** |
| --- | --- |
| 1. Justify the use of mixed methods | Introduction and Methods (Study design). Clinician adoption of AI-CDSS involves both measurable attitudes (survey) and contextual reasoning about trust, workflow, and design (interviews). Neither strand alone captures both the magnitude and the mechanisms of adoption barriers. |
| 2. Describe the design: purpose, priority, sequence | Methods (Study design). Convergent (concurrent triangulation) design; the survey and interviews were conducted in parallel (February to April 2025) and given equal priority (QUAN+QUAL). |
| 3. Describe each method: sampling, data collection, analysis | Methods (Recruitment and participants; Survey procedures; Interview procedures; Data analysis). Survey: N=57, descriptive and inferential statistics with 95% Wilson CIs. Interviews: n=11, six-phase thematic analysis (Braun and Clarke) with dual coding and consensus adjudication. |
| 4. Describe where and how integration occurred, and who participated in it | Methods (Study design; Data analysis) and Results (Table 2). Integration occurred at the methods level through shared constructs across the survey and interview guide, and at the interpretation level through a joint display assigning integration types (convergent, complementary, expansion, emergent) by consensus between the two coders. |
| 5. Describe limitations of one method associated with the presence of the other | Limitations. The survey constrains depth (fixed items, small subgroups); the interviews constrain breadth (n=11, ICU-weighted, five-provider case scenario). The A/B preferences arise from the interview strand and are exploratory. |
| 6. Describe insights gained from mixing that would not have been gained separately | Results (Table 2) and Discussion. Examples: interviews explain the survey training barrier through prior rollout experiences; the AI-branding fatigue finding is emergent from interviews alone and motivates the de-branding discussion; the acuity-dependent trust gradient (survey) is explained by clinicians' insistence on human judgment in high-acuity care (interviews). |
