# Supplementary material for: Clinician Trust and Human Factors in AI-Enabled Clinical Decision Support in Acute Care: Mixed Methods Study
Source: JMIR Hum Factors. 2026 Oct 2;13:e95472. doi: 10.2196/95472 (PMC13633065; doi:10.2196/95472)
Supplement: Supplementary file 11 [file humanfactors-v13-e95472-s014.docx]

Framework: Eysenbach G. J Med Internet Res. 2004 Sep 29;6(3):e34.

| **CHERRIES Item Category** | **Checklist Item** | **Reporting** |
| --- | --- | --- |
| Design | Describe survey design | This study used a cross-sectional, exploratory online survey as part of a mixed-methods human factors evaluation. The target population was clinicians involved in direct patient care in ICU and ED settings at Emory Healthcare. The survey used a convenience sample of eligible clinicians recruited through listservs, flyers, departmental presentations, and word-of-mouth referral. |
| IRB approval and informed consent | IRB approval | The study was approved by the Emory University Institutional Review Board and Indiana University Institutional Review Board. |
|  | Informed consent | Participants reviewed written study information and completed electronic consent before accessing the survey. Consent materials described the study purpose, voluntary participation, survey length of approximately 10-15 minutes, risks/benefits, compensation, confidentiality, REDCap data storage, data sharing with Indiana University for analysis, investigator contact information, and participant rights. |
|  | Data protection | Survey and consent data were collected and stored in REDCap. Access was restricted to authorized study team members. Identifying information collected for consent and study administration was not used in analysis or reporting. Contact information for gift card distribution was collected through a separate form and was not linked to survey responses. Results were reported in aggregate. |
| Development and pre-testing | Development and testing | The survey instrument was developed through an iterative consensus process with subject matter experts in critical care and health informatics. Before deployment, the REDCap survey was reviewed by the study team for content, usability, and technical functionality. |
| Recruitment process and sample access | Open survey versus closed survey | Targeted survey, but not password-protected. Distributed to ICU and ED clinicians through listservs, flyers with QR codes, departmental presentations, and word-of-mouth referral. Because QR links could be accessed through open-ended channels, the exact access denominator could not be determined. |
|  | Contact mode | Initial contact occurred through a combination of electronic and in-person/offline recruitment approaches, including email listservs, QR-coded flyers, departmental presentations, and word-of-mouth referral. |
|  | Advertising the survey | The survey was advertised through invitation emails distributed via ICU and ED provider listservs at Emory Healthcare hospitals, IRB-approved flyers posted in clinical units, presentations during departmental meetings, and word-of-mouth referrals among clinical staff. Flyers included a QR code directing interested individuals to the online REDCap survey platform. |
| Survey administration | Web/E-mail | The survey was administered electronically through REDCap. Survey responses were captured automatically in the REDCap database. |
|  | Context | The survey was not hosted on a public website. It was administered through REDCap and distributed to clinicians in ICU and ED settings within Emory Healthcare. |
|  | Mandatory/voluntary | Participation was voluntary. Participants could choose to complete the survey, participate in an interview, or both, and could discontinue participation at any time. |
|  | Incentives | Survey participants received a $10 gift card after survey completion. Interview participants received a $25 gift card after interview completion. |
|  | Time/Date | The survey was open from February 9, 2025, to April 1, 2025. |
|  | Randomization of items or questionnaires | Survey items were not randomized. |
|  | Adaptive questioning | Branching logic was used based on response to a survey question so that applicable follow-up items were displayed. The main survey domains were otherwise presented in a fixed order. |
|  | Number of items | The survey contained 32 survey items across demographics, AI-CDSS use, general AI perceptions, trust in AI, usability features, and one open-ended item. This count excludes consent items and the separate gift card/contact form. |
|  | Number of screens/pages | Survey questions were distributed across 6 REDCap pages. These followed the study information/written consent and electronic consent/signature instruments. |
|  | Completeness check | REDCap required-field validation was enabled for most structured survey items, as indicated by “must provide value” prompts. Participants could discontinue participation at any time. |
|  | Review step | A separate final review page was not included. Participants completed the REDCap survey pages and submitted responses at the end of the survey. |
| Response rates | Unique site visitor | Unique site visitor counts were not available. The survey was not hosted on a public website with unique visitor tracking. |
|  | View rate | A view rate could not be calculated because the number of unique individuals who viewed the survey invitation or survey landing page was not available. |
|  | Participation rate | Participation rate could not be precisely calculated. Recruitment materials were distributed through ICU and ED listservs including approximately 500 clinicians, plus flyers, presentations, and word-of-mouth referral; therefore, the exact denominator was uncertain. |
|  | Completion rate | Fifty-eight respondents initiated the survey. Fifty-seven completed the survey, yielding a 98.2% completion rate. One partially incomplete response was not included in the final analytic dataset.. |
| Preventing multiple entries | Cookies used | Cookies were not used by the study team to identify or prevent duplicate entries. |
|  | IP check | IP addresses were not used by the study team to identify or exclude duplicate entries. |
|  | Log file analysis | Formal log file analysis was not used. REDCap records were manually reviewed for potential duplicates using participant name/consent information. |
|  | Registration | Participants were not required to create a username or log in to complete the REDCap survey. |
| Analysis | Handling of incomplete questionnaires | Only completed survey responses were included in the final analysis. Responses with incomplete survey submission were excluded from the analytic dataset. |
|  | Questionnaires submitted with an atypical timestamp | No exclusion criteria based on survey completion time or atypical timestamps were applied. |
|  | Statistical correction | No weighting, propensity score adjustment, or other statistical correction was applied to adjust for the non-probability convenience sample. |
