# Supplementary material for: Clinician Trust and Human Factors in AI-Enabled Clinical Decision Support in Acute Care: Mixed Methods Study
Source: JMIR Hum Factors. 2026 Oct 2;13:e95472. doi: 10.2196/95472 (PMC13633065; doi:10.2196/95472)
Supplement: Supplementary file 12 [file humanfactors-v13-e95472-s015.docx]

Framework: Tong A, Sainsbury P, Craig J. Int J Qual Health Care. 2007;19(6):349-357.

| **COREQ item** | **Response / location** |
| --- | --- |
| Domain 1: Research team and reflexivity |  |
| 1. Interviewer/facilitator | Interviews were conducted by the lead analyst (MD). Stated in Methods (Interview procedures). |
| 2. Credentials | MD: MS in Health Informatics. Dentist BDS |
| 3. Occupation | MD: Graduate Research Assistant |
| 4. Gender | Female |
| 5. Experience and training | Worked as a dentist, followed by training in Health Informatics, including qualitative research. |
| 6. Relationship established prior to study | External research student at Emory University. Primary affiliation at Indiana University. |
| 7. Participant knowledge of the interviewer | The survey included a consent form outlining the study purpose and procedures. Those who expressed interest in interviews were followed up to schedule a convenient time. Prior to beginning each interview, the interviewer (MD) introduced herself, described her role as a graduate research assistant and student at Indiana University, and external researcher at Emory University, and reiterated the study goals and consent procedures before proceeding |
| 8. Interviewer characteristics | Reflexivity Statement (Methods): lead analyst had prior knowledge of AI-CDSS design principles; several team members are PRECISE trial investigators. |
| Domain 2: Study design |  |
| 9. Methodological orientation and theory | Thematic analysis per Braun and Clarke six-phase framework; deductive coding from the interview guide plus inductive coding (Methods, Data analysis). |
| 10. Sampling | Convenience/volunteer sampling of survey completers who opted into interviews (Methods, Recruitment). |
| 11. Method of approach | Listserv emails, QR-coded flyers, departmental presentations, word of mouth (Methods, Recruitment). |
| 12. Sample size | n=11 (6 nurses, 3 physicians, 2 APPs), reported in Results. |
| 13. Non-participation | Non-participation was not formally tracked; interviews were conducted with all participants who opted in and responded to follow-up contact. |
| 14. Setting of data collection | Zoom videoconference (Methods, Interview procedures). |
| 15. Presence of non-participants | Only the primary researcher and participant were present during the interview |
| 16. Description of sample | Clinical role reported in Results; department mix in Results (Survey findings) for the parent sample. |
| 17. Interview guide | Structured guide developed from literature; provided in Appendix 7; pilot testing was done with other clinically-trained Health Informatics students. |
| 18. Repeat interviews | None conducted. |
| 19. Audio/visual recording | Audio recorded via Zoom (Methods). |
| 20. Field notes | Field notes were taken during observations prior to participant interviews, and later after the interview before coding. |
| 21. Duration | Approximately 30 to 45 minutes (Methods). |
| 22. Data saturation | Saturation reached after the tenth interview; information power (Malterud) and code saturation thresholds (Hennink et al) cited (Results, Interview findings). |
| 23. Transcripts returned to participants | NO |
| Domain 3: Analysis and findings |  |
| 24. Number of data coders | Two coders (MD, KC) with a third reviewer (SP) adjudicating (Methods, Data analysis). |
| 25. Description of the coding tree | 36 themes from ~690 codes consolidated into 6 clusters; full structure in Appendix 8. |
| 26. Derivation of themes | Both deductive (interview guide) and inductive (emergent patterns) (Methods). |
| 27. Software | ATLAS.ti and Microsoft Excel (Methods). |
| 28. Participant checking | Systematic member-checking during analysis (Reflexivity Statement). Participants were employed at Emory Healthcare and verified at the start. |
| 29. Quotations presented | Representative quotations with participant identifier and clinical role throughout Results and Appendix 8. |
| 30. Data and findings consistent | Joint display (Table 2) links quotations and survey estimates to each interpretation. |
| 31. Clarity of major themes | Six thematic clusters presented as Results subsections. |
| 32. Clarity of minor themes | Sub-themes and diverse/contradictory perspectives in Appendix 13; critical views quoted in Results. |
